# Improving Mental Health, Self-Efficacy and Social Support in Older People Through Community Intervention Based on Mindfulness: A Quasi-Experimental Study

**DOI:** 10.3390/healthcare14020229

**Published:** 2026-01-16

**Authors:** Denis Juraga, Darko Roviš, Mihaela Marinović Glavić, Lovorka Bilajac, Maša Antonić, Hein Raat, Vanja Vasiljev

**Affiliations:** 1 Department of Social Medicine and Epidemiology, Faculty of Medicine, University of Rijeka, 51000 Rijeka, Croatia; darko.rovis@medri.uniri.hr (D.R.); mihaela.marinovic@uniri.hr (M.M.G.); lovorka.bilajac@uniri.hr (L.B.); vanjav@uniri.hr (V.V.); 2 Teaching Institute of Public Health of Primorje-Gorski Kotar County, 51000 Rijeka, Croatia; 3 Department of Public Health, Faculty of Health Studies, University of Rijeka, 51000 Rijeka, Croatia; 4 Department of Microbiology and Parasitology, Faculty of Medicine, University of Rijeka, 51000 Rijeka, Croatia; masa.antonic@medri.uniri.hr; 5 Department of Public Health, Erasmus MC-University Medical Centre Rotterdam, 3015 Rotterdam, The Netherlands; h.raat@erasmusmc.nl

**Keywords:** mental health, self-management, self-efficacy, chronic disease, mindfulness, public health, social support, aged

## Abstract

**Background:** Aging is a complex process that involves various biological, psychological and social changes. Moreover, older people (≥65 years) are more susceptible to lower self-efficacy and social support, as well as deteriorating mental health. As the global population ages, there is a growing demand for evidence-based interventions tailored to address specific mental health problems, enhance social support and improve overall well-being. The aim of this study was to investigate the effectiveness of a seven-week mindfulness-based community intervention on mental health, self-efficacy and social support in older people. **Methods:** This quasi-experimental nonrandomized study included 257 participants who were divided into an intervention group and a comparison group that did not participate in the seven-week mindfulness-based community intervention and was not part of a waiting list. Assessments were conducted before the intervention began and 6 months after its completion. **Results:** The results revealed a significant reduction in depression in the intervention group (*p* < 0.001). Furthermore, the intervention led to a significant improvement in general self-efficacy, chronic disease self-management self-efficacy, physical activity and nutritional self-efficacy compared with the comparison group. Perceived social support increased within the intervention group; however, covariate adjusted between-group effects for social support were not statistically significant. **Conclusions:** Overall, the mindfulness-based community intervention was associated with improvements in current depressive symptoms and multiple self-efficacy domains at 6-month follow-up in older people in a community setting. Effects on perceived social support were less robust, and no statistically significant between-group differences were observed after adjustment for baseline covariates. The results of the present study show that this program leads to immediate health benefits in terms of mental health and self-efficacy in older people while contributing to the development of effective strategies for chronic disease self-management.

## 1. Introduction

Aging is multifaceted and complex, encompassing a variety of biological, psychological and social changes that are part of the experience of growing older [1]. Examples include vulnerability to stress, loss of self-efficacy, mental health problems, and lack of social support (often compounded by age-related issues such as physical health problems and cognitive decline) [1]. Given the growing age structure of the global population, attention to the mental well-being of older people will become a critical factor in the provision of evidence-based interventions in the future that consider the well-being and quality of life of older people. The term “older people” conceptually denotes individuals in later life for whom age-related changes in health, functional capacity, and social roles may influence mental health and the management of chronic conditions [2]. Operationally, in line with commonly used thresholds in health research and policy, this study defines older people as persons aged 65 years or older [3].

Mental health is an essential component of general health. It encompasses emotional, psychological and social well-being and enables individuals to successfully cope with the complexities and challenges of life [4]. A healthy mind is characterized by maintaining fulfilling relationships, coping with stress, feeling satisfied and coping with change or difficulties [5]. Maintaining mental health is particularly important for older people who are struggling with age-related problems such as cognitive decline, physical difficulties and social isolation [6]. Numerous factors, including biological factors such as genetic predispositions and changes in brain chemistry, influence the onset and progression of mental health problems in older people [7]. In addition, psychological factors such as personality traits and coping mechanisms play important roles in how people respond to stress and adversity [8]. Understanding and managing these various factors that influence the mental health of older people is becoming increasingly important as the world’s population ages. Older people often suffer from mental health problems such as depression and anxiety. According to research data, 20% of people over the age of 60 suffer from mental health problems [9]. Furthermore, older people’s mental health problems are often not properly recognized and treated, exacerbating the impact on individuals and society as a whole [10].

In addition to mental health, non-communicable chronic diseases (NCDs), as a physical component of health, are the main cause of disability and mortality in older people [9]. The most common diseases are cardiovascular diseases; cancer; and respiratory diseases such as chronic obstructive pulmonary disease (COPD) and type II diabetes. According to the World Health Organization, NCDs are responsible for 71% of all deaths worldwide, which corresponds to approximately 41 million deaths per year [11]. Cardiovascular diseases (CVDs), particularly heart disease and stroke, are the leading causes of death worldwide. In 2019, they were responsible for an estimated 17.9 million deaths, representing 32% of all deaths worldwide, with the majority occurring in low- and middle-income countries [12]. Type II diabetes (T2D) is another major NCD with a profound global impact. According to the International Diabetes Federation, approximately 463 million adults (aged 20–79 years) had some form of diabetes (type I or type II, diagnosed or undiagnosed) in 2019. This number is expected to rise to 700 million by 2045 [13]. Chronic diseases significantly impact the quality of life of older people, affecting mobility, well-being and mental health [14,15,16]. To cope with this burden, older people can acquire new life skills to effectively self-manage their chronic diseases [17,18]. Self-efficacy is a psychological concept that refers to a person’s confidence in their ability to perform a particular task or behavior [19]. This important element influences motivation, perseverance and resilience and is associated with several positive outcomes, including better stress management, improved mental health and higher life satisfaction [20]. A strong sense of self-efficacy can help older people overcome the obstacles of aging, maintain their independence, adapt to changing roles and responsibilities, maintain a good quality of life and adopt health-promoting behaviors [21,22]. This is related to the concept of successful aging, which is characterized by the preservation of physical health, cognitive function and mental well-being and is closely linked to self-efficacy [23,24].

One of the possible approaches to support successful aging could be mindfulness, which has been shown to improve emotional regulation and reduce stress, which is crucial for maintaining self-efficacy later in life [25]. Mindfulness, as a state of mind characterized by nonjudgmental observation of the present moment [26], has attracted much attention for its potential to promote mental health, resilience and social relationships. Mindfulness practices such as meditation, body scans and mindfulness exercises such as yoga have their roots in ancient Buddhist philosophy and aim to improve awareness of the present moment, resulting in psychological and physical benefits [27,28]. Studies consistently show that mindfulness practices improve psychological well-being by reducing symptoms of depression, anxiety and stress [29] while improving self-compassion, life satisfaction, emotional regulation [30] and health-related quality of life [31]. In terms of stress reduction, mindfulness promotes adaptive coping mechanisms such as problem solving, emotion control and cognitive reorganization, which help people better manage stress and respond to stressors [32]. Mindfulness practices are also associated with increased self-efficacy by promoting greater self-awareness, self-compassion and a more accurate understanding of one’s own abilities and limitations [33]. In addition, mindfulness-based interventions (MBIs), including structured programs such as Mindfulness-based stress reduction (MBSR) and Mindfulness-based cognitive therapy (MBCT), have been increasingly evaluated in older people. Recent studies have provided evidence that suggests that mindfulness interventions are associated with beneficial effects on mental health and well-being later in life, including outcomes related to depressive symptoms and psychological distress [34,35]. Evidence has also accumulated for MBIs in cardiometabolic populations relevant to this study. A systematic review and a meta-analysis of the cardiovascular disease adult population indicated that MBIs have favorable effects on psychological and physiological outcomes among adults with CVDs [36]. In diabetes, a systematic review and meta-analysis of randomized controlled trials reported improvements in mental health outcomes (stress, anxiety) and reductions in HbA1c [37].

Despite this growing literature, few studies have examined mindfulness-based programs delivered pragmatically through community and primary care pathways for older people with cardiovascular disease and/or type 2 diabetes, and there remains uncertainty about which psychosocial targets (e.g., depressive symptoms, domain-specific self-efficacy, and perceived social support) change most reliably in real-world community delivery and whether such changes are maintained at medium-term follow-up.

### 1.1. Study Aim

The aim of this study was to evaluate the effectiveness of a seven-week mindfulness-oriented community intervention, compared with a nonrandomized comparison group, in improving depressive symptoms, multiple self-efficacy domains (general, chronic disease self-management, physical activity and nutritional self-efficacy), and perceived social support among people aged ≥65 years living with cardiovascular disease and/or type II diabetes, as assessed at baseline (T0) and at the six-month follow-up (T1). This seven-week mindfulness-based workshop program combines the theoretical framework of salutogenesis, the person-centered approach, positive psychology, the theory of behavior change based on the Transtheoretical Model, mindfulness, the GROW coaching model (Goal, Reality, Options, and Will model) and two already known evidence-based programs: the Chronic Disease Self-Management Program (CDSMP) and the Mindfulness-based Living Course [38,39,40].

### 1.2. Research Questions

1. Among people aged ≥ 65 years with CVD and/or T2D, does participation in mindfulness-based community intervention lead to a greater reduction in depressive symptoms from baseline to follow-up than in the comparison group?
2. Does the mindfulness-based community intervention lead to greater improvements from baseline to follow-up in self-efficacy, assessed across (a) general self-efficacy, (b) chronic disease self-management self-efficacy, (c) physical activity self-efficacy, and (d) nutritional self-efficacy, compared with the comparison group?
3. Does the mindfulness-based community intervention lead to greater improvement in perceived social support from baseline to follow-up compared with the comparison group?

## 2. Materials and Methods

### 2.1. Participants

The participants were part of a quasi-experimental design for prospective evaluation of interventions, which included the comparison of those receiving the intervention with those not receiving it. On this basis, the analysis includes the estimation of the difference in the amount of change over time in the outcome of interest between the two groups, beginning with the intervention and moving forward in time [41]. This quasi-experimental nonrandomized study was conducted from 1 October 2019 to 15 August 2022, during which the study participants were divided into two groups. The first consisted of the participants who took part in a mindfulness-based intervention, and the second was the comparison group. The participants were residents of the city of Rijeka and the wider urban area. Inclusion in each group was based on the geographical location of the participants and their general practitioners (GPs)/community patronage nurses. The participating practices served populations from similar urban neighborhoods, and as indicated later in the Results section, baseline comparisons revealed no significant differences between groups in terms of age, education, or other socioeconomic indicators, suggesting limited systematic socioeconomic differences between the catchment areas, although unmeasured contextual variation in neighborhood characteristics or service accessibility cannot be excluded. Patients who met the inclusion criteria were enrolled consecutively, as they expressed interest upon arrival at the GPs or community patronage nurses (first come, first served), who informed them about the project, its activities, and the responsibilities associated with participation in the intervention or comparison group until study group-specific targets were reached. Randomization was not feasible because the mindfulness-based program was implemented as a community service delivered at specific sites linked to participating GP practices and community patronage nurses, and group-based workshops required scheduling and capacity planning within existing primary care/community infrastructures. The participants were therefore allocated pragmatically on the basis of their registration with GP practices.

The intervention and comparison groups were recruited and assessed during the same calendar period, with follow-up assessments conducted at comparable times relative to baseline, ensuring that both groups were exposed to the same overall COVID-19 pandemic phase and public health context. Pandemic-related restrictions were defined at the national/regional level and applied uniformly across the study area; therefore, differential restrictions between groups were unlikely.

Owing to the community-based nature of the project, our sample size was determined by the number of older people available and willing to participate during enrollment. To enhance methodological rigor, a power analysis was conducted to assess the adequacy of the sample. For the total sample (*n* = 257), a power of approximately 80% was obtained to establish a moderate effect size (Cohen’s d ~ 0.5) for the main results at a significance level of 0.05.

### 2.2. Inclusion/Exclusion Criteria

The inclusion criteria for the participants were individuals of both sexes, who were older than 65 years, who were suffering from chronic diseases such as cardiovascular diseases/conditions (heart failure, hypertension) and/or type II diabetes, who lived in the city of Rijeka and the wider urban area and were able to participate in the study for a period of 6 months. The exclusion criteria, on the other hand, were persons who do not have a permanent residence in the city of Rijeka and the wider urban area, persons who were unable to participate in the study for 6 months (persons planning a longer period or terminally ill patients), persons who are homeless, persons suffering from a mental illness according to the Diagnostic and Statistical Manual of Mental Disorders, Fourth Edition (DSM IV), persons with cognitive impairments and people with alcohol and other substance addiction.

### 2.3. Methods

The intervention participants attended the seven-week workshop program (one workshop per week). The first workshop, “Mind and Body Training for Your Own Wellbeing”, introduced participants to the skill of mindfulness and its impact on emotional and social intelligence as well as the development of empathy and compassion. The participants also gained insights into the process of coping with stress and how to recognize autopilots in everyday life, as well as their own protective or risk factors. “Healthy Habits”, the second workshop, aimed to encourage participants to be more aware of their lifestyle. The facilitator of the workshop introduced them to the model of habit change phases and explained how a person can overcome their own resistance and stress in the process of habit change. Mindfulness practices (3-min breathing and body scan) were presented and carried out as a foundation for maintaining awareness of one’s thoughts, feelings, body sensations and surroundings. The content of the third workshop, “Healthy Mindset”, was based on two main types of mindsets: fixed and growing [42]. The workshop sought to encourage participants to develop a growth-oriented mindset that can help them focus on personal success with the support of self-compassion and the 5-4-3-2-1 (grounding) mindfulness practice. In the fourth workshop, “Healthy Eating”, the participants were introduced to the concepts of mindful eating and healthy eating with regard to their health. They practiced the mindful eating technique and the 5-4-3-2-1 (grounding) mindfulness practice during the session, with a focus on integrating these practices into their daily routine. The fifth workshop, “Healthy Physical Activity”, focuses on the importance and impact of daily physical activity on health. In addition, the participants were introduced to the skill of setting SMART goals (Specific, Measurable, Achievable, Relevant and Time-Bound). So-called smart goals are tools that can positively influence the development of ideas, guide individuals toward the productive use of time and resources and increase the chances of achieving their own life goals [43]. The participants were also taught to practice mindful walking. The sixth workshop, “Healthy Relationships”, emphasized healthy relationships as one of the key components of health and personal well-being. In addition, participants were introduced to the concept of emotional intelligence as the ability to manage and express one’s own emotions and recognize those of others. To support this workshop, participants practiced the mindfulness exercise of loving kindness, silently repeating phrases of warmth and goodwill to cultivate compassion for themselves and others. The content of the seventh workshop, “Healthy Living Despite Chronic Disease”, aimed to encourage participants to take control of their lives by actively engaging in the management of their health. Through the practice of mindfulness (3-min breathing and self-compassion break), participants were encouraged to focus their attention on their own resources to reduce the impact of chronic disease on health and quality of life rather than focusing all their attention on the symptoms of the disease. By promoting the self-management process, participants acquired the ability to adopt healthy lifestyle habits, such as a healthy diet and physical activity. On the other hand, the participants assigned to the comparison group did not partake in the mindfulness workshops and continued with their routine care and daily activities. In addition, they were not offered the opportunity to join the mindfulness program after the study was completed. The program was guided by an integrated theoretical framework that combined salutogenesis (Antonovsky’s sense of coherence) [44], person-centered care [45], positive psychology (Seligman’s PERMA model) [46], behavior change theory based on the Transtheoretical Model [47], coaching principles from the GROW model [48], and elements from two established evidence-based programs (CDSMP and the Mindfulness-Based Living Course). These frameworks informed the structure of the sessions (e.g., focus on meaningful goals and coping resources), facilitation style (nonjudgmental, collaborative, participant-centered) and emphasis on enhancing internal resources for well-being rather than solely addressing disease or deficits. A detailed description of how each framework was operationalized within the SEFAC workshop program is provided in the Supplementary Materials.

The workshops were organized such that the participants were divided into smaller groups of up to 10 people. These studies lasted 2 h and considered all epidemiological measures that were active during the Coronavirus disease 2019 (COVID-19) pandemic. While participation in the program required attendance at the scheduled sessions, session attendance and home-practice adherence were not systematically captured at the individual level for reporting or statistical analysis in this manuscript.

The workshops were delivered by trained facilitators, i.e., certified mindfulness trainers and psychologists, who had prior experience working with older people in community settings and had completed formal training in mindfulness-based interventions (Mindfulness-Based Living Course) [40]. All the facilitators followed a structured SEFAC workshop manual to standardize content and sequence across groups. Fidelity was supported through the use of this manual and regular team meetings in which facilitators reviewed session plans and discussed implementation challenges; no formal session-by-session fidelity checklist or audio/video rating was used. At the end of each workshop, the participants received a standardized home-practice sheet specifying brief daily mindfulness exercises (e.g., 3-min breathing space, body scan, mindful walking; approximately 10–20 min per day) and behavior-change tasks (e.g., setting, monitoring and revising SMART goals for physical activity, healthy eating and other lifestyle behaviors) to complete between sessions. The participants were invited to reflect on their home practice experiences at the beginning of the subsequent session. The aim of the seven-week workshop program was to bring about behavioral changes in the participants, make them aware of their habits and lifestyle, and reduce the risk factors that are modifiable: obesity, unhealthy diet, physical inactivity, harmful alcohol consumption, tobacco use and learning new skills to improve self-efficacy, self-esteem and the ability to self-manage their health. In addition, the workshop program was designed to support participants in developing resilience skills and to help reduce stress, anxiety, and depression [49].

To assess the medium-term effects and equalize the burden on participants, a 6-month follow-up was conducted. A 6-month window is commonly used in community-based interventions, including chronic disease self-management programs and mindfulness workshops with older people, and provides a comparable measure of the persistence of change in the outcomes assessed [50,51].

### 2.4. Outcome Measures

To assess the effectiveness of the intervention in terms of mental health (the presence of current depressive symptoms), general self-efficacy, chronic disease self-management self-efficacy, physical activity, nutritional self-efficacy and social support, a section of the SEFAC questionnaire was used. The questionnaire was developed as part of the SEFAC project [52]. To assess internal consistency, Cronbach’s alphas were calculated with acceptable values equal to or greater than 0.60 [53]. To assess the presence of current depressive symptoms, the 8-item Patient Health Questionnaire Depression Scale (PHQ-8) (Cronbach’s alpha 0.78) was used, with scores ranging from 0–24, with higher scores indicating greater severity of depressive symptoms [54]. Self-efficacy was assessed via four questionnaires: the General Self-Efficacy Scale (GSES) (Cronbach’s alpha 0.90), the Self-Efficacy for Managing Chronic Diseases 6-item Scale (SEMCD) (Cronbach’s alpha 0.92), the Physical Exercise Self-Efficacy Scale (PESES) (Cronbach’s alpha 0.96) and the Nutrition Self-Efficacy Scale (NSES) (Cronbach’s alpha 0.96) [12,55,56]. Higher scores were associated with higher levels of general self-efficacy, chronic disease self-management self-efficacy, physical activity and nutritional self-efficacy. Social support was assessed via the Oslo Social Support Scale (OSSS-3) (Cronbach’s alpha 0.60). The total score ranges from 3 to 14, with high scores indicating a high level of social support and low scores indicating a low level of social support [57,58]. Four separate self-efficacy domains were assessed to capture both general confidence in one’s ability to cope with demands (general self-efficacy) [59,60,61] and more specific beliefs related to chronic disease self-management [62], physical activity [63], and healthy nutrition [64]. These domains are particularly relevant in older people with cardiovascular disease and/or type 2 diabetes, for whom daily self-management, maintaining an active lifestyle, and adhering to dietary recommendations are central to long-term outcomes [65].

To summarize, the SEFAC questionnaire in this study combines several validated instruments (PHQ-8, GSES, SEMCD, PESES, NSES and OSSS-3). Loneliness and social isolation were not directly assessed as separate constructs; instead, perceived social support was measured via the Oslo Social Support Scale (OSSS-3).

In addition to the outcome measures, the following general sociodemographic characteristics were also collected: age, sex, presence of chronic diseases and conditions, marital status, household composition, education level and household income.

### 2.5. Statistical Analysis

The data were collected at two points in time: T0 (baseline measurement) and T1 (measurement 6 months after the end of the intervention). The collected data were statistically analyzed via IBM SPSS Statistics 28.0.0.0. (IBM Corporation, Armonk, NY, USA). All participants who completed the baseline questionnaire and the follow-up questionnaire were included in the final analysis. The data were analyzed via a mixed ANOVA model including both within-subject (time) and between-subject (group) factors. This approach accounted for participants’ repeated measures over time and directly tested the interaction between group (intervention vs. comparison) and time (pre-intervention and 6-month follow-up). By using mixed ANOVA with an appropriate covariance structure, it was possible to better model individual trajectories and group differences, improving the rigor of the analysis. To analyze the effect size, the partial eta squared was also calculated, which indicates the proportion of variance in the dependent variable that is attributable to a particular factor [66]. The interpretation of partial eta squared values follows the thresholds proposed by Jacob Cohen (1988): small effect (η^2^ ≈ 0.01), medium effect (η^2^ ≈ 0.06) and large effect (η^2^ ≈ 0.14) [67]. The level of statistical significance was α = 0.05.

Baseline differences between the intervention and comparison groups were examined via χ^2^ tests for categorical variables and *t*-tests or nonparametric tests for continuous variables depending on the normality of the data. Variables that differed significantly between groups at baseline (sex, marital status, household composition, and baseline physical activity self-efficacy, where relevant) were included as covariates in the mixed ANOVA models to reduce confounding by these imbalances. For each outcome, mixed ANOVA with time (baseline and follow-up) as the within-subject factor and group (intervention vs. comparison) as the between-subject factor was performed. We reported *p* values, partial eta squared (η^2^) as a measure of effect size for interaction terms, and 95% confidence intervals (CIs) for pre–post changes within each group. Statistical significance was set at *p* < 0.05.

To address baseline imbalances inherent to the nonrandomized design, we conducted a series of analysis of covariance (ANCOVA) models as sensitivity analyses for each outcome. For current depressive symptoms (PHQ-8), general self-efficacy (GSES), chronic disease self-management self-efficacy (SEMCD), physical exercise self-efficacy (PESES), nutritional self-efficacy (NSES), and perceived social support (OSSS-3), the follow-up score at T1 was specified as the dependent variable, and the study arm (intervention vs. comparison) was specified as the fixed factor. The baseline (T0) value of the respective outcome was entered as a covariate, together with sociodemographic variables that differed between groups at baseline (sex, marital status, and household composition). Because physical exercise self-efficacy differed between groups at baseline, baseline PESES (PESES_T0) was additionally included as a covariate in all ANCOVA models. Adjusted group differences at follow-up were evaluated via the F statistic with a two-sided significance level of *p* < 0.05, and adjusted means were used to support interpretation of between-group differences after baseline adjustment.

For the outcomes reported in this study, all 257 participants had complete data at both T0 and T1. The mixed ANOVA models therefore used complete-case data without any imputation. Participants with missing values for a given outcome would have been excluded from that specific analysis, but this situation did not occur for the scales included in this study.

**Figure 1:**
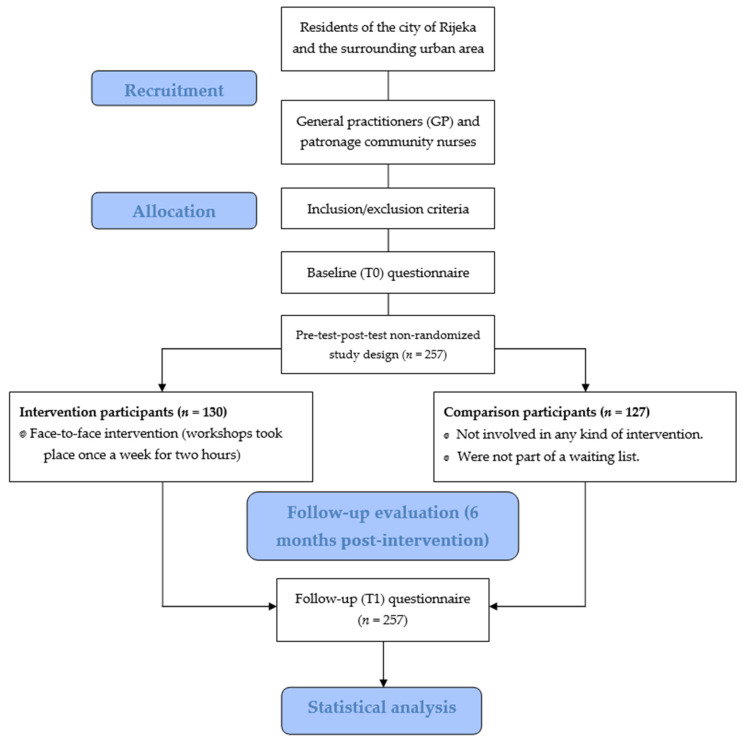
Flow diagram of the study.

Figure 1 shows the flow of the study, from the recruitment of participants to the follow-up evaluation.

### 2.6. Ethical Considerations

This research was conducted in accordance with all applicable policies designed to ensure the proper conduct of the research and the safety of the persons participating in this research while respecting the principles of good clinical practice. The research respected fundamental ethical and bioethical principles—personal integrity (autonomy), justice, charity and innocence—in accordance with the Nuremberg Code and the latest revision of the Declaration of Helsinki of the World Medical Association, the Health Act of the Republic of Croatia (OG 158/08, 71/10, 139/10, 22/11, 84/11, 12/12, 35/12, 70/12, 82/13, 100/18 and 125/19), the Act on the Rights of Patients of the Republic of Croatia (OG 169/04, 37/08) and Regulation (EU) 2016/679 of the European Parliament and of the Council of 27 April 2016, on the protection of individuals regarding the processing of personal data and on the free movement of such data.

The study was approved by the Ethics Committee of the University of Rijeka, Faculty of Medicine (Class: 003-08/20-01/91; Registration number: 2170-24-09-8-20-3) and by the Ethics Committee of the Health Centre of Primorje-Gorski Kotar County, Rijeka, Croatia (Registration number: 01-47/2-2-21).

The study was registered on 30 August 2018 under ISRCTN registry number ISRCTN11248135 on the UK’s Clinical Study Registry, c/o BMC, The Campus, 4 Crinan Street, London, N1 9XW, United Kingdom.

The reporting of this pre-test–post-test nonrandomized study was guided by the Transparent Reporting of Evaluations with Nonrandomized Designs (TREND) Statement Checklist [68].

## 3. Results

A total of 257 participants were included in the study: 130 in the intervention group and 127 in the comparison group. The general sociodemographic characteristics and baseline primary outcome measures for both study groups are shown in Table 1.

As shown in Table 1, the average age of the participants who took part in the mindfulness-based intervention was 71.9 years, and that of the participants in the comparison group was 72.1 years (*p* = 0.173), with the age range extending from 65–88 years. Regarding the presence of chronic non-communicable diseases, almost equal numbers of participants from both groups had heart failure, hypertension, or type II diabetes, with no statistically significant differences. The differences in education level and household income between the intervention and comparison participants were also not significant (*p* = 0.494 and *p* = 0.339, respectively). To better visualize the results, household income was divided into five deciles. On the other hand, a statistically significant difference in sex structure between these two groups was demonstrated (*p* < 0.001). In the intervention group, almost 90% of the participants were women, whereas 65.4% were women in the comparison group. A significant difference was also evident in the marital status of the participants (*p* = 0.008). More than 80% of the participants in the comparison group lived with others (family, friends), whereas 36.9% (*n* = 48) of the participants in the intervention group lived alone.

As shown in Table 2, which presents the primary outcome measures at baseline (T0) for the intervention and comparison groups, there were no statistically significant differences between participants in the intervention and comparison groups regarding the presence of current depressive symptoms, general self-efficacy, chronic disease self-management self-efficacy, nutritional self-efficacy, or social support. On the other hand, there was a statistically significant difference between the observed groups in terms of physical activity self-efficacy (*p* = 0.006).

At baseline, the intervention and comparison groups did not differ significantly in terms of age, education, household income or chronic disease indicators (Table 1). However, significant differences were observed in sex distribution, marital status and household composition, as well as in physical activity self-efficacy (Table 1 and Table 2). These variables were therefore included as covariates in the mixed ANOVA models examining intervention effects.

To account for baseline group differences, a series of ANCOVAs were conducted for current depressive symptoms (PHQ-8), general self-efficacy (GSES), chronic disease self-management self-efficacy (SEMCD), physical exercise self-efficacy (PES), nutritional self-efficacy (NSES), and social support (OSSS-3) at follow-up (T1). In each model, the follow-up score was entered as the dependent variable; group (face-to-face intervention vs. comparison) was entered as the fixed factor; and the corresponding baseline score (T0) together with sex, marital status, household composition, and baseline physical exercise self-efficacy (PESES_T0) were entered as covariates.

After adjustment, the intervention group presented significantly lower current depressive symptoms at follow-up than did the comparison group did (adjusted means: 4.72 vs. 5.93; F (1,250) = 12.53, *p* < 0.001), independent of baseline current depressive symptoms, which was a strong predictor of follow-up current depressive symptoms (F (1,250) = 110.32, *p* < 0.001). No sociodemographic covariates were significantly associated with current depressive symptoms.

Similarly, the intervention group demonstrated significantly greater general self-efficacy (GSES) at follow-up than did the comparison group did (adjusted means: 33.67 vs. 30.87; F (1,250) = 21.99, *p* < 0.001). Baseline general self-efficacy was a strong predictor of follow-up general self-efficacy cores (F (1,250) = 47.25, *p* < 0.001), and higher baseline physical exercise self-efficacy (PESES_T0; F (1,250) = 10.42, *p* = 0.001) and female sex (F (1,250) = 7.27, *p* = 0.008) were independently associated with higher follow-up general self-efficacy.

For chronic disease self-management self-efficacy (SEMCD), adjusted analyses again indicated higher follow-up scores in the intervention group than in the comparison group (adjusted means: 7.73 vs. 6.86; F (1,250) = 21.69, *p* < 0.001), with baseline chronic disease self-management self-efficacy emerging as the primary predictor (F (1,250) = 71.25, *p* < 0.001). No demographic covariates were significant.

Comparable intervention effects were observed for physical exercise self-efficacy (PESES; adjusted means: 14.26 vs. 12.45; F (1,251) = 12.68, *p* < 0.001) and nutritional self-efficacy (NSES; adjusted means: 14.38 vs. 12.63; F (1,250) = 14.80, *p* < 0.001), with baseline levels of the respective outcomes consistently predicting follow-up scores (all *p* < 0.001). The baseline PESES_T0 score was also independently associated with the NSES score at follow-up (F (1,250) = 4.998, *p* = 0.026).

In contrast, no significant group difference was observed for social support (OSSS-3) at follow-up after adjustment (adjusted means: 10.17 vs. 9.94; F (1,250) = 0.95, *p* = 0.332). Follow-up social support was primarily predicted by baseline social support (F (1,250) = 115.46, *p* < 0.001).

At the baseline measurement (T0), there were no significant differences in current depressive symptoms between participants in the intervention and comparison groups (MD = 0.455, SE = 0.402, 95% CI [−0.336, 1.246], *p* = 0.258). However, six months after the second measurement (T1), a significant difference emerged, with the intervention group reporting significantly lower levels of depressive symptoms than the comparison group did (MD = −0.954, SE = 0.409, 95% CI [−1.758, −0.149], *p* = 0.020).

A repeated-measures ANOVA, controlling for sex, marital status, household composition, and baseline physical activity self-efficacy, revealed no significant main effect for changes in depressive symptoms from T0 to T1 [F (1,251) = 1.687, *p* = 0.195, η^2^ = 0.007], suggesting that there was no overall significant difference across all participants irrespective of group affiliation. Additionally, no significant main effects were found for depressive symptoms based on group affiliation [F (1,251) = 0.488, *p* = 0.485, η^2^ = 0.002], sex [F (1,251) = 0.140, *p* = 0.709, η^2^ = 0.001], marital status [F (1,251) = 0.023, *p* = 0.881, η^2^ = 0.000], or household composition [F (1,251) = 0.083, *p* = 0.774, η^2^ = 0.000]. However, the analysis revealed a significant interaction between current depressive symptoms and baseline Physical Exercise Self-Efficacy Scale (PESES) scores [F (1,251) = 9.141, *p* = 0.003, η^2^ = 0.035], indicating that changes in current depressive symptoms significantly varied depending on baseline physical activity self-efficacy scores (small to medium effect size; η^2^ = 0.035). Participants with higher physical activity self-efficacy levels at baseline showed greater improvements (reduction) in current depressive symptoms over time than those with lower physical activity self-efficacy. Moreover, there was a significant interaction effect between time and group affiliation, indicating that the type of intervention influenced changes in current depressive symptoms between T0 and T1 [F (1,251) = 13.537, *p* < 0.001, η^2^ = 0.051], with a medium-sized effect (η^2^ = 0.051). Detailed ANOVA results for current depressive symptoms are provided in Table 3.

Table 4 shows that there were significant differences in the reported levels of current depressive symptoms between the first and second time points of measurement in the intervention group of participants. The participants who participated in the mindfulness-based interventions experienced a significant decrease in current depressive symptoms, whereas the comparison group of participants experienced no significant change.

At the baseline measurement (T0), no significant differences in general self-efficacy levels were found between the intervention and comparison groups (MD = −0.200, SE = 0.626, 95% CI [−1.433, 1.033], *p* = 0.750). However, six months after the intervention (T1), the intervention group presented significantly greater general self-efficacy than did the comparison group did (MD = 2.722, SE = 0.651, 95% CI [1.440, 4.004], *p* < 0.001).

A repeated-measures ANOVA, controlling for sex, marital status, household composition, and baseline physical activity self-efficacy, indicated no significant main effect for changes in general self-efficacy between T0 and T1 [F (1,251) = 0.392, *p* = 0.532, η^2^ = 0.002], suggesting no overall significant difference across all participants. However, there was a significant main effect of group affiliation on the general level of self-efficacy, regardless of the time of measurement [F (1,251) = 5.577, *p* = 0.019, η^2^ = 0.022], which indicates a small effect size, whereas belonging to the intervention or comparison group had a sustained overall effect, not just a time-limited effect. No significant main effects were found for sex [F (1,251) = 3.222, *p* = 0.074, η^2^ = 0.013], marital status [F (1,251) = 1.810, *p* = 0.180, η^2^ = 0.007], household composition [F (1,251) = 0.365, *p* = 0.546, η^2^ = 0.001], or baseline Physical Exercise Self-Efficacy Scale (PESES) scores [F (1,251) = 0.184, *p* = 0.668, η^2^ = 0.001]. A significant interaction effect was observed between time and group affiliation, indicating that the type of intervention influenced changes in general self-efficacy between the two measurement points [F (1,251) = 17.400, *p* < 0.001, η^2^ = 0.065], with a medium-sized effect. Detailed ANOVA results for general self-efficacy levels are provided in Table 5.

Table 6 shows that there were significant differences in the levels of general self-efficacy between the first and second time points of measurement in participants who took part in the mindfulness-based workshops (*p* < 0.001), as well as in the comparison group participants (*p* = 0.023). The intervention participants experienced a significant increase in the level of general self-efficacy. On the other hand, the comparison group participants reported significantly lower levels of general self-efficacy.

At baseline (T0), no significant differences were found in chronic disease self-management self-efficacy between the intervention and comparison groups (MD = 0.036, SE = 0.203, 95% CI [−0.364, 0.436], *p* = 0.858). However, at six months post-intervention (T1), participants in the intervention group reported significantly greater chronic disease self-management self-efficacy than did those in the comparison group did (MD = 0.884, SE = 0.210, 95% CI [0.470, 1.298], *p* < 0.001).

A repeated-measures ANOVA, adjusting for sex, marital status, household composition, and baseline physical activity self-efficacy, revealed no significant main effect for changes in chronic disease self-management self-efficacy from T0 to T1 [F (1,251) = 1.225, *p* = 0.269, η^2^ = 0.005], suggesting no overall significant difference regardless of group. Nonetheless, a significant main effect was observed for group affiliation across measurement points [F (1,251) = 6.734, *p* = 0.010, η^2^ = 0.026], indicating that a small effect size, which means belonging to the intervention or comparison group, had a sustained overall effect, not just a time-limited effect. No significant main effects were found regarding sex [F (1,251) = 0.084, *p* = 0.772, η^2^ = 0.000], marital status [F (1,251) = 0.937, *p* = 0.334, η^2^ = 0.004], or household composition [F (1,251) = 0.564, *p* = 0.453, η^2^ = 0.002]. Additionally, significant interaction effects emerged between baseline Physical Exercise Self-Efficacy Scale (PESES) scores and chronic disease self-management self-efficacy [F (1,251) = 5.787, *p* = 0.017, η^2^ = 0.023] and between time and group affiliation [F (1,251) = 15.872, *p* < 0.001, η^2^ = 0.059]. The former indicates that higher baseline PESES scores significantly influenced changes in higher chronic disease self-management self-efficacy overall (small effect size), whereas the latter suggests that the type of intervention significantly influenced these changes between T0 and T1 (medium effect size). The detailed ANOVA results are provided in Table 7.

Table 8 shows that there are significant differences in the levels of chronic disease self-management self-efficacy between the first and second measurement time points for the participants who participated in the mindfulness-based workshops (*p* < 0.001), whereas there is no significant difference between the two measurement time points for the participants in the comparison group (*p* = 0.470).

Significant differences in physical activity self-efficacy were found at baseline (T0) between participants in the intervention and comparison groups (MD = 1.455, SE = 0.580, 95% CI [0.313, 2.597], *p* = 0.013). This difference persisted six months after the intervention (T1), with the intervention group again reporting significantly greater physical activity self-efficacy than the comparison group did (MD = 2.563, SE = 0.584, 95% CI [1.413, 3.713], *p* < 0.001).

A repeated-measures ANOVA, adjusting for sex, marital status, and household composition, revealed no significant main effect for changes in physical activity self-efficacy between T0 and T1 [F (1, 252) = 0.222, *p* = 0.638, η^2^ = 0.001], suggesting no overall significant difference in self-efficacy over time irrespective of group. However, there was a significant main effect for group affiliation across both measurement points [F (1, 252) = 15.744, *p* < 0.001, η^2^ = 0.059], indicating a medium effect size, which means that belonging to the intervention or comparison group had a sustained overall effect, not just a time-limited effect. No significant main effects were found for sex [F (1, 252) = 0.288, *p* = 0.592, η^2^ = 0.001], marital status [F (1, 252) = 1.814, *p* = 0.179, η^2^ = 0.007], or household composition [F (1, 252) = 0.361, *p* = 0.549, η^2^ = 0.001]. Additionally, a significant interaction effect between time and group affiliation was observed, with differences in self-efficacy changes over time depending on the intervention type [F (1, 252) = 3.731, *p* = 0.055, η^2^ = 0.015], although with a small effect size. Detailed ANOVA results for physical activity self-efficacy are presented in Table 9.

Table 10 shows that there were significant differences between the first and second measurement time points in physical activity self-efficacy levels among the participants who took part in the mindfulness-based workshops (*p* = 0.024), whereas there were no significant changes in the participants in the comparison group (*p* = 0.576). Mindfulness-based workshop participants reported significantly higher levels of physical activity self-efficacy, whereas there were no significant changes in the comparison group participants across the two measurement time points.

At the baseline measurement (T0), there was no significant difference in nutritional self-efficacy between participants in the intervention and comparison groups (MD = 0.017, SE = 0.512, 95% CI [−0.993, 1.026], *p* = 0.974). However, six months later, at the second measurement (T1), participants in the intervention group reported significantly greater nutritional self-efficacy than did those in the comparison group did (MD = 1.755, SE = 0.499, 95% CI [0.772, 2.738], *p* < 0.001).

A repeated-measures ANOVA, controlling for sex, marital status, household composition, and baseline physical activity self-efficacy, revealed a significant overall improvement in nutritional self-efficacy from T0 to T1 among all participants, regardless of group affiliation [F (1,251) = 8.617, *p* = 0.004, η^2^ = 0.033], with a small to medium effect size. The analysis further revealed several notable effects: first, a sustained overall effect of group affiliation (intervention vs. comparison) on nutritional self-efficacy across both measurement points [F (1,251) = 4.325, *p* = 0.039, η^2^ = 0.017, small effect]; second, a significant interaction between sex and nutritional self-efficacy changes over time [F (1,251) = 5.612, *p* = 0.019, η^2^ = 0.022, small effect], suggesting differences between men and women in how their nutritional self-efficacy improved; third, a significant interaction with baseline physical activity self-efficacy scores, indicating that participants with higher initial physical activity self-efficacy had greater gains in nutritional self-efficacy [F (1,251) = 4.715, *p* = 0.031, η^2^ = 0.018, small effect]; and finally, a significant interaction effect between time and group affiliation, confirming that the type of intervention significantly influenced the changes in nutritional self-efficacy from T0 to T1 [F (1,251) = 10.142, *p* = 0.002, η^2^ = 0.039, small to medium effect]. No significant effects were observed for marital status [F (1,251) = 0.318, *p* = 0.573, η^2^ = 0.001] or household composition [F (1,251) = 1.826, *p* = 0.178, η^2^ = 0.007]. Detailed ANOVA results for nutritional self-efficacy levels are presented in Table 11.

The results presented in Table 12 show that there are significant differences in the level of nutritional self-efficacy between the first and second measurement time points in the participants who took part in the interventions (*p* = 0.008). Additionally, there was a significant difference between the T0 and T1 measurements in the comparison group of participants (*p* = 0.045). The intervention participants experienced a significant increase in the level of nutritional self-efficacy. On the other hand, the comparison group participants reported significantly lower levels of nutritional self-efficacy at the two time points.

No significant differences in social support levels were observed between the intervention and comparison groups at the baseline measurement (T0) (MD = −0.488, SE = 0.281, 95% CI [−1.042, 0.066], *p* = 0.084) or at six months post-intervention (T1) (MD = −0.047, SE = 0.289, 95% CI [−0.617, 0.523], *p* = 0.870).

A repeated-measures ANOVA, controlling for sex, marital status, household composition, and baseline physical activity self-efficacy, revealed no significant main effects for changes in social support between T0 and T1 [F (1,251) = 1.660, *p* = 0.199, η^2^ = 0.007]. Similarly, no significant interactions were found between social support and sex [F (1,251) = 0.297, *p* = 0.587, η^2^ = 0.001], marital status [F (1,251) = 0.931, *p* = 0.335, η^2^ = 0.004], household composition [F (1,251) = 0.822, *p* = 0.366, η^2^ = 0.003], or baseline PESES scores [F (1,251) = 1.418, *p* = 0.235, η^2^ = 0.006]. Additionally, there were no significant effects based on group affiliation across both measurement points [F (1,251) = 2.716, *p* = 0.101, η^2^ = 0.011], nor was there an interaction effect between time and group affiliation [F (1,251) = 1.125, *p* = 0.290, η^2^ = 0.004]. Detailed ANOVA results for social support levels are provided in Table 13.

Although the interaction effect between group and time (OSSS-3 * Category) in Table 13 was not statistically significant (*p* = 0.101), the pairwise comparisons in Table 14 revealed that participants in the intervention group experienced a significant increase in social support from T0 to T1, whereas no such change was observed in the comparison group. This suggests that the intervention may have had a positive effect within the intervention group, even if the overall interaction did not reach statistical significance. One possible explanation for this discrepancy is that the average change in social support between the groups was not large enough to reach significance in the overall model, possibly due to limited statistical power. In addition, the groups were not completely equivalent at the start of the study, as the comparison group had a slightly higher OSSS-3 score, which may have limited the scope for improvement. High within-group variability or small effect sizes could also mask the interaction effects between group and time, even if there is a true within-group effect.

## 4. Discussion

The aim of this study was to investigate the effectiveness of a seven-week mindfulness-based community intervention on mental health (the presence of current depressive symptoms), four types of self-efficacy (general self-efficacy, chronic disease self-management self-efficacy, physical activity self-efficacy and nutritional self-efficacy) and the social support domain in older people compared with a comparison group. The aim of the seven-week mindfulness-based community intervention was to stimulate behavioral change in both older people at risk of developing chronic non-communicable diseases and those suffering from them, to make them aware of their own habits and lifestyles and to encourage them to acquire new skills to improve their self-efficacy in managing their health and thus increase their quality of life. The results of this study indicate significant improvements in mental health, self-efficacy and social support in the intervention group compared with the comparison group. As shown in the previous section, six measurement scales were used, one for mental health and social support and four for self-efficacy, and the intervention group performed better on each of these scales after the intervention period. The comparison group, on the other hand, performed slightly worse or almost equal in each individual category after the completion of the intervention period.

Among all participants in both the intervention and comparison groups, the majority were women (89.2% and 65.4%, respectively). Lower male participation is common in public health interventions and similar research. According to the data from the literature, several factors may explain the lower interest of older men in participating in public health interventions: the habit of using health services less frequently than women do; the self-perception that they are less prone to health problems and therefore less likely to participate in preventive health measures or community interventions; social norms and stereotypes about masculinity, according to which participation in these or similar activities is seen as a sign of weakness; a lower willingness to adopt healthier lifestyles; a greater propensity to engage in riskier behaviors; and a general neglect of their own health [69,70,71,72,73,74]. In the more recent literature, there are many studies on public health in which a larger proportion of participants were women [75,76,77,78,79]. The available literature states that the unequal proportion of men and women in studies may affect the final result, but conducting statistical analyses allows for the creation of a “representative sample” [80]. The aforementioned challenges of involving men in research should also be anticipated, and a solution needs to be found in relation to involving a greater number of male participants in health promotion interventions. Among the included intervention participants, more than 60% live in a marriage or other type of union. Notably, people who have a wider network of social interactions and are more active in the community are more satisfied with their lives and their position in society [81].

To reduce the impact of baseline imbalances, we adjusted all mixed ANOVA models for variables that differed significantly between groups at baseline (sex, marital status, household composition and baseline physical activity self-efficacy where relevant). Nonetheless, statistical adjustment cannot fully eliminate the possibility of residual confounding.

### 4.1. Mental Health

The results of our study revealed that older people in the intervention group experienced a significant decrease in current depressive symptoms (from 5.9 to 4.9; *p* < 0.001), whereas the current depressive symptom scores in the comparison group did not change significantly over the follow-up period (*p* = 0.255). Consistent with this pattern, the covariate-adjusted repeated-measures ANOVA revealed a statistically significant time × group interaction with a small-to-moderate effect (F (1,251) = 13.537, *p* < 0.001, η^2^ = 0.051), indicating that the pre–post change differed between groups. In practical terms, the mean reduction of approximately one PHQ-8 point represents a modest symptom-level improvement [82]. However, in an older population, even modest shifts in depressive symptom burden may be relevant at the population level, particularly when these shifts are achieved through a brief, community-delivered program. This result is also consistent with previous studies that have shown the effectiveness of mindfulness-based interventions in reducing depressive symptoms in at-risk populations, which undoubtedly include the older population [83]. A retrospective analysis by Young and Baime (2010) also revealed the positive effects of mindfulness on the mental health of older people and reported lower rates of anxiety and depression [84]. Mindfulness-based interventions have been shown to improve emotion regulation and increase positive affect, thereby contributing to an overall improvement in mental health [85]. As mentioned, some studies emphasize the importance of emotion regulation, a process that influences the experience and expression of emotions and overall well-being and thus represents a mediator between mindfulness practices and the reduction in symptoms of depression [86]. Additionally, participants with higher physical activity self-efficacy levels at baseline presented greater reductions in current depressive symptoms over time than did those with lower physical activity self-efficacy levels. This finding is consistent with studies that show that individuals with greater self-efficacy for physical activity are more likely to exercise, which can lead to a reduction in depressive symptoms. Conversely, those with low self-efficacy may find it difficult to initiate or maintain regular exercise, which may increase depressive symptoms [87,88,89].

### 4.2. General Self-Efficacy

Our study revealed that participants in the intervention group achieved higher levels of general self-efficacy (*p* < 0.001), chronic disease management self-efficacy (*p* < 0.001), physical activity self-efficacy (*p* = 0.024) and nutritional self-efficacy (*p* = 0.008) after the intervention. On the other hand, the comparison group participants reported lower levels of general (*p* = 0.023) and nutritional self-efficacy (*p* = 0.045), whereas the other types of self-efficacy did not change significantly over the follow-up period. Notably, at the baseline (T0) measurement, there was a statistically significant difference between the observed groups in terms of physical activity self-efficacy (*p* = 0.006). These results could be partly interpreted by the difference in the timing of consent to participate in the interventions, considering the epidemiological situation due to the COVID-19 pandemic and the participants’ motivation to participate in the intervention. The results for the intervention participants are consistent with those of previous studies demonstrating the positive impact of mindfulness on self-efficacy in different populations [90,91]. General self-efficacy refers to the concept that determines how a person feels, thinks, motivates and behaves and is considered the primary segment for changing certain behaviors [20]. In addition, there is also a relationship between self-efficacy and the practice of mindfulness, which is consequently associated with greater satisfaction and quality of life [92,93]. Higher levels of self-efficacy affect the ability to cope with stressful situations and control emotional states, problem solving, the presence of optimism and positive thoughts in everyday life and the belief in the ability to achieve set goals [20,94,95]. As this research was conducted during the COVID-19 pandemic, the participants in the face-to-face intervention likely acquired coping skills through workshops and the practice of mindfulness, not only in dealing with stressful situations on a daily level but also with stressors caused by the pandemic. On the other hand, six months after the first measurement, participants in the comparison group presented significantly lower levels of general self-efficacy, indicating the possible influence of stressful situations on daily functioning. The results obtained also emphasize the probable need to adopt resistance mechanisms to maintain well-being and general health, which was not the case for the participants in the comparison group, as they did not take part in the intervention.

### 4.3. Chronic Disease Self-Management Self-Efficacy

Chronic disease self-management self-efficacy refers to a person’s ability to manage their health condition, i.e., to control fatigue, physical discomfort or pain, emotional difficulties, and other activities related to managing chronic diseases and conditions [96]. This can be achieved by practicing mindfulness via three mechanisms: attention control, emotion regulation and self-awareness [97]. Individuals with higher levels of self-efficacy in coping with chronic diseases have higher levels of resilience in overcoming challenges associated with chronic diseases, as demonstrated by previously conducted face-to-face-delivered mindfulness-based programs [98].

### 4.4. Physical Activity and Nutritional Self-Efficacy

According to the results of this study, a significantly greater level of physical activity self-efficacy and nutritional self-efficacy was demonstrated in the intervention group than in the control group between the baseline (T0) and follow-up measurements. Data from the literature show that mindfulness has been shown to be an effective tool for lifestyle change and the adoption of healthier lifestyle habits [99]. Mindfulness has a positive effect on the adoption of healthy lifestyle habits, such as physical activity. It improves attention, promotes a nonjudgmental attitude and increases an individual’s willingness to accept negative feelings and experiences [100,101,102,103]. The combined influence of these factors promotes the development of self-regulation and self-efficacy, leading to an increase in the ability to maintain adopted habits despite the possible occurrence of physical discomfort (e.g., breathlessness) or self-limiting thoughts (e.g., “I will never be healthy even if I am physically active”). With respect to the willingness to change one’s eating habits; mindfulness has also proven to be effective [104,105]. In studies examining nutritional self-efficacy, women tended to report higher levels of self-efficacy for healthy eating and dietary choices than men did. This suggests that women may have more confidence in their ability to maintain healthy eating habits and make good dietary choices, have greater awareness of nutritional information, a stronger focus on health, or a greater perceived susceptibility to health consequences from poor diet [106,107].

Across self-efficacy outcomes, the intervention group demonstrated consistent improvements from baseline to follow-up, whereas the comparison group showed either no improvement or small decreases. Taken together, the effect sizes (η^2^ ≈ 0.039–0.065 for the domains with significant interactions) indicate small-to-moderate intervention effects, which is consistent with effect magnitudes commonly reported for brief, group-based community self-management and mindfulness interventions [108,109] and supports the practical relevance of targeting multiple self-efficacy domains central to chronic disease management and behavioral changes in older people [60].

### 4.5. Social Support

With regard to perceived social support, the interpretation requires nuance. Although the intervention group showed a statistically significant pre–post increase in OSSS-3 scores, the covariate-adjusted repeated-measures model did not demonstrate a statistically significant time × group interaction, and the baseline-adjusted ANCOVA likewise indicated no statistically significant between-group difference at follow-up. Therefore, unlike depressive symptoms and observed self-efficacy outcomes, the present data do not support a conclusion that the program increased perceived social support relative to the comparison group over the follow-up period, and this outcome should be interpreted cautiously. Several factors may account for this pattern. First, OSSS-3 is a brief instrument that may have limited sensitivity for detecting small changes over time [108]. Second, social support and social network characteristics may change more slowly than intrapersonal outcomes, such as mood and perceived self-efficacy, and a longer follow-up period may be needed to observe robust between-group differences [109,110]. Finally, pandemic-related constraints on social contact during the study period may have limited opportunities for broader changes in participants’ social interactions beyond the intervention setting, possibly attenuating observable between-group effects on a brief support measure [111]. Mindfulness and social support are inextricably linked [112,113], especially in difficult times such as the COVID-19 pandemic [114,115]. From a mechanistic perspective, mindfulness-based interventions may plausibly influence perceived social support indirectly by strengthening personal capacities that shape how individuals engage with and appraise available social resources [116]. Mindfulness practice may reduce stress reactivity and negative affect [117] and support emotion regulation and self-compassion [112], which could facilitate more constructive communication, greater openness to receiving help, and a more adaptive appraisal of social support [118]. However, these pathways remain inferential in the present study: we did not measure interpersonal processes (e.g., communication quality, group cohesion, help-seeking behavior) and the adjusted models did not demonstrate statistically robust between-group differences in perceived social support. Future studies should therefore use more comprehensive social support instruments and assess hypothesized interpersonal mediators to clarify whether, how, and under what conditions mindfulness-based community programs translate into measurable improvements in perceived social support.

Considering the results in the intervention group, it is reasonable to assume that this mindfulness-based community intervention was successful because it provided participants with a sufficient level of psychological support to overcome this stressful and very challenging time, especially with respect to their mental health and corresponding current depressive symptoms. In addition to the mental health benefits of the intervention, it has also been shown to be an effective tool for improving the health of participants with chronic diseases by encouraging them to self-manage and self-monitor their disease and/or condition, empowering them and increasing their self-efficacy, as well as adopting a healthier lifestyle in relation to modifiable factors to achieve better health outcomes. With respect to perceived social support, scores increased within the intervention group; however, adjusted between-group analyses did not demonstrate a statistically significant effect, and social support outcomes should therefore be interpreted cautiously. These and similar studies could also provide evidence for health policy makers to propose reforms for more effective and efficient models of chronic disease prevention and management.

### 4.6. Limitations of the Study

The present study has several limitations that should be addressed. These limitations are not unique to this study but also apply to similar studies in the field. One major limitation is potential selection bias, as this study used a quasi-experimental, nonrandomized design with allocation on the basis of GP practice/geographical area and enrollment on a ‘first come, first served’ basis. This approach may have introduced selection bias and residual confounding. Although we adjusted our analyses for baseline differences between groups, unmeasured factors such as motivation, perceived health, or preexisting social connectedness may have influenced both participation and outcomes. Therefore, the observed associations should be interpreted with caution and not as definitive causal effects. This limits the generalizability of the results to the overall population.

Another important aspect is the demographic imbalance between the intervention and comparison groups. Despite similar group sizes, there was a clear sex gap, with the intervention group being predominantly female and containing very few male participants. This discrepancy could be due to factors such as differences in gender interest in health-related behaviors, as women generally show greater interest in healthier lifestyles, whereas men engage in riskier health behaviors [72,119,120,121]. In addition, cultural factors specific to the Croatian social context, where traditional gender roles may influence participation, may have played a role. Furthermore, the sample homogeneity from a single geographical area does not fully represent the broader older population group, and the findings of this research should be generalized with caution. Future studies should engage more diverse groups of older people from wider areas to verify that the benefits of mindfulness-based community interventions are consistent across different subpopulations and regions.

As the allocation of participants to a particular study group was not randomized, only limited clear causal conclusions can be drawn. The inclusion of a comparison group strengthens the study, as it provides a benchmark for comparison, but the lack of random allocation means that alternative explanations for the observed differences cannot be completely ruled out.

A further limitation is that session attendance, home-practice adherence, and formal fidelity monitoring (e.g., structured checklists or independent ratings) were not systematically documented; therefore, we could not quantify intervention exposure or assess dose–response relationships.

Because the study took place during the COVID-19 pandemic, contextual factors such as restrictions on social contact, changes in access to primary care and community services, and pandemic-related stress could have influenced mental health and social outcomes in both groups. Although both groups were followed during the same period, we cannot fully exclude the possibility that time-varying restrictions or behavioral adaptations affected exposure to the intervention (e.g., attendance) or routine care and thus contributed to the observed changes.

Importantly, differences in self-efficacy in terms of physical activity and, to a lesser extent, nutrition between the two groups were noted. The participants in the intervention group showed greater self-efficacy at baseline, which was probably due to their greater interest in a healthier lifestyle. These factors should be considered when interpreting the results, as they may have influenced the results obtained in this study.

Although this study included a six-month follow-up period, it may not have been sufficient to demonstrate long-term effects. Six months is a modest follow-up period, so longer-term effects remain unknown. Future studies with longer follow-up periods should be conducted to determine whether the improvements observed are sustainable or whether participants may need regular “booster” sessions to maintain benefits over a longer period of time. Long-term sustainability is critical for public health impacts, and this is an important direction for future research.

The findings concerning social support should be interpreted with caution because of the OSSS-3’s limited reliability. The OSSS-3’s brevity (only 3 items) often yields lower alpha values in general, and this trade-off was made to reduce respondent burden for our older participants. Short scales often have difficulties reaching high Cronbach’s alpha values, as this internal consistency factor depends on the number of questionnaire items [108]. The lower internal consistency might attenuate observed effects or mask true associations, thus potentially underestimating the intervention’s impact on social support. Future research should consider the use of more comprehensive social support scales to improve reliability.

## 5. Conclusions

Mindfulness-based community intervention that combines the theoretical framework of salutogenesis, the person-centered approach, positive psychology, the theory of behavior change based on the Transtheoretical Model, mindfulness, the GROW coaching model and two evidence-based programs (Chronic Disease Self-Management Program (CDSMP) and the Mindfulness-based Living Program) was associated with improved outcomes in current depressive symptoms and multiple self-efficacy domains among older people at the six-month follow-up. These findings support the potential value of targeting psychological resources and self-management confidence through structured, community-delivered programs. The positive impact was particularly notable given that a large part of the study was conducted at the height of the COVID-19 pandemic. This shows that the proposed approach can be effective even under very unfavorable conditions.

Effects on perceived social support were less robust. Although social support increased within the intervention group, adjusted between-group analyses did not demonstrate a statistically significant difference at follow-up. Therefore, conclusions regarding social support should be interpreted cautiously, and future evaluations should use more comprehensive measures and assess hypothesized interpersonal mechanisms (e.g., group cohesion, mindful communication, help-seeking behaviors).

The results of the study suggest that this approach can have a direct benefit on the health of the older population and that it can be useful in the prevention and/or management of chronic diseases as well as in the development of various self-management strategies. Although this seven-week intervention with a six-month follow-up showed promising results, future research should include larger and more diverse samples, multi-site implementation, prospective monitoring of attendance and fidelity, and extended follow-up beyond six months to determine durability of effects and to examine whether changes in self-efficacy and mental health translate into improvements in objective health indicators, self-management behaviors, and healthcare utilization.

Overall, mindfulness-based interventions have the potential for further study and the search for valid ways to manage the health of an increasingly aging population.

## Figures and Tables

**Table 1:** General sociodemographic characteristics of the intervention and comparison groups.

	Intervention Group (*n* = 130) *n* (%)	Comparison Group (*n* = 127) *n* (%)	*p*
$\mathrm{Age}\;(y)\;(\bar{x})$	71.9	72.1	0.173
Sex			
Women	116 (89.2)	83 (65.4)	**<0.001**
Men	14 (10.8)	44 (34.6)	
Chronic diseases			
Heart failure	16 (12.3)	17 (13.4)	0.853
Hypertension	74 (56.9)	71 (55.9)	0.900
Type II diabetes	22 (16.9)	26 (20.5)	0.523
Marital status			
Single	16 (12.3)	13 (10.2)	**0.008**
Partnership	65 (50.0)	92 (72.4)	
Widow(er)	49 (37.7)	22 (17.3)	
Household composition			
Living alone	48 (36.9)	22 (17.3)	**<0.001**
Living with others	82 (63.1)	105 (82.7)	
Educational level			
Primary or less	24 (18.5)	8 (6.3)	0.494
Secondary	64 (49.2)	84 (66.1)	
Tertiary or higher	42 (32.3)	35 (27.6)	
Household income			
Decile 1 + 2 (<239–345 €)	24 (18.5)	10 (7.9)	0.339
Decile 3 + 4 (346–597 €)	50 (38.5)	55 (43.3)	
Decile 5 + 6 (598–889 €)	26 (20.0)	39 (30.7)	
Decile 7 + 8 (890–1.287 €)	20 (15.4)	19 (15.0)	
Decile 9 + 10 (1.288–>1.606 €)	10 (7.7)	4 (3.1)	

*n* = number of participants; % = percentage; y = years; $\bar{x}$ = mean; *p* value = level of marginal significance within a statistical hypothesis test, representing the probability of the occurrence of a given event; € = euro.

**Table 2:** Baseline primary outcome measures for the intervention and comparison groups.

	Intervention Group (*n* = 130) $\bar{x}$ (σ)	Comparison Group (*n* = 127) $\bar{x}$ (σ)	*p*
Mental health (current depressive symptoms)			
PHQ-8	5.9 (3.0)	5.6 (3.4)	0.508
Self-efficacy			
GSES	31.8 (4.8)	32.0 (4.9)	0.829
SEMCD	7.1 (0.1)	6.9 (0.2)	0.317
PESES	13.8 (4.4)	12.2 (4.3)	**0.006**
NSES	13.9 (4.2)	12.9 (4.3)	0.061
Social support			
OSSS-3	9.54 (2.2)	9.91 (2.1)	0.166

$\bar{x}$ = mean; σ = standard deviation; *p* value = level of marginal significance within a statistical hypothesis test, representing the probability of the occurrence of a given event; PHQ-8 = 8-item Patient Health Questionnaire Depression Scale; GSES = General Self-Efficacy Scale; SEMCD = Self-Efficacy for Managing Chronic Diseases 6-item Scale; PESES = Physical Exercise Self-Efficacy Scale; NSES = Nutrition Self-Efficacy Scale; OSSS-3 = Oslo Social Support Scale.

**Table 3:** Analysis of variance (ANOVA) with repeated measures related to current depressive symptoms.

	Sum of Squares	df	Mean Square	F	*p*	η^2^
PHQ-8	6.863	1	6.863	1.687	0.195	0.007
Group: intervention, comparison	6.902	1	6.902	0.488	0.485	0.002
PHQ-8 × Sex	0.569	1	0.569	0.140	0.709	0.001
PHQ-8 × Marital status	0.092	1	0.092	0.023	0.881	0.000
PHQ-8 × Household composition	0.337	1	0.337	0.083	0.774	0.000
PHQ-8 × PESES_T0	37.180	1	37.180	9.141	**0.003**	0.035
PHQ-8 × Group: intervention, comparison	55.063	1	55.063	13.537	**<0.001**	0.051
Error (PHQ-8)	1020.953	251	4.068			
Error (Group: intervention, comparison)	3549.590	251	14.142			

PHQ-8 = 8-item Patient Health Questionnaire Depression Scale; PESES_T0 = Physical Exercise Self-Efficacy Scale score at baseline; df = degrees of freedom; F = the ratio of the mean square for the between groups divided by the mean square within groups; *p* = level of marginal significance within a statistical hypothesis test, representing the probability of the occurrence of a given event; η^2^ = partial eta squared.

**Table 4:** Differences in the expressed levels of current depressive symptoms between the first and second measurement time points depending on group affiliation.

Participants per Intervention	PHQ-8 (T0) $\bar{x}$ (σ)	PHQ-8 (T1) $\bar{x}$ (σ)	MD	SE	*p*	95% Confidence Interval for Difference	
						Lower Bound	Upper Bound
Intervention group (*n* = 130)	5.9 (3.0)	4.9 (2.9)	1.109	0.260	**<0.001**	0.597	1.620
Comparison group (*n* = 127)	5.6 (3.4)	5.8 (3.4)	−0.300	0.263	0.255	−0.818	0.218

PHQ-8 = 8-item Patient Health Questionnaire Depression Scale; T0 = baseline measurement; T1 = follow-up measurement; $\bar{x}$ = mean; σ = standard deviation; *n* = number of participants; MD = mean difference between the observed groups; SE = standard error of the mean difference between the observed groups; *p* = level of marginal significance within a statistical hypothesis test, representing the probability of the occurrence of a given event.

**Table 5:** Analysis of variance (ANOVA) with repeated measures related to general self-efficacy.

	Sum of Squares	df	Mean Square	F	*p*	η^2^
GSES	5.336	1	5.336	0.392	0.532	0.002
Group: intervention, comparison	176.508	1	176.508	5.577	**0.019**	0.022
GSES × Sex	43.865	1	43.865	3.222	0.074	0.013
GSES × Marital status	24.639	1	24.639	1.810	0.180	0.007
GSES × Household composition	4.972	1	4.972	0.365	0.546	0.001
GSES × PESES_T0	2.510	1	2.510	0.184	0.668	0.001
GSES × Group: intervention, comparison	236.892	1	236.892	17.400	**<0.001**	0.065
Error (GSES)	3417.285	251	13.615			
Error (Group: intervention, comparison)	7943.533	251	31.648			

GSES = General Self-Efficacy Scale; PESES_T0 = Physical Exercise Self-Efficacy Scale score at baseline; df = degrees of freedom; F = the ratio of the mean square for the between groups divided by the mean square within groups; *p* = level of marginal significance within a statistical hypothesis test, representing the probability of the occurrence of a given event; η^2^ = partial eta squared.

**Table 6:** Differences in expressed levels of general self-efficacy between the first and second measurement time points depending on group affiliation.

Participants per Intervention	GSES (T0) $\bar{x}$ (σ)	GSES (T1) $\bar{x}$ (σ)	MD	SE	*p*	95% Confidence Interval for Difference	
						Lower Bound	Upper Bound
Intervention group (*n* = 130)	31.8 (4.8)	33.6 (5.3)	−1.821	0.475	**<0.001**	−2.757	−0.886
Comparison group (*n* = 127)	32.0 (4.9)	30.9 (4.9)	1.101	0.481	**0.023**	0.153	2.048

GSES = General Self-Efficacy Scale; T0 = baseline measurement; T1 = follow-up measurement; $\bar{x}$ = mean; σ = standard deviation; *n* = number of participants; MD = mean difference between the observed groups; SE = standard error of the mean difference between the observed groups; *p* = level of marginal significance within a statistical hypothesis test, representing the probability of the occurrence of a given event.

**Table 7:** Analysis of variance (ANOVA) with repeated measures related to chronic disease self-management self-efficacy.

	Sum of Squares	df	Mean Square	F	*p*	η^2^
SEMCD	1.539	1	1.539	1.225	0.269	0.005
Group: intervention, comparison	23.506	1	23.506	6.734	**0.010**	0.026
SEMCD × Sex	0.106	1	0.106	0.084	0.772	0.000
SEMCD × Marital status	1.177	1	1.177	0.937	0.334	0.004
SEMCD × Household composition	0.709	1	0.709	0.564	0.453	0.002
SEMCD × PESES_T0	7.271	1	7.271	5.787	**0.017**	0.023
SEMCD × Group: intervention, comparison	19.943	1	19.943	15.872	**<0.001**	0.059
Error (SEMCD)	315.374	251	1.256			
Error (Group: intervention, comparison)	876.170	251	3.491			

SEMCD = Self-Efficacy for Managing Chronic Diseases 6-item Scale; PESES_T0 = Physical Exercise Self-Efficacy Scale score at baseline; df = degrees of freedom; F = the ratio of the mean square for the between groups divided by the mean square within groups; *p* = level of marginal significance within a statistical hypothesis test, representing the probability of the occurrence of a given event; η^2^ = partial eta squared.

**Table 8:** Differences in the expressed levels of chronic disease self-management self-efficacy between the first and second measurement time points depending on group affiliation.

Participants per Intervention	SEMCD (T0) $\bar{x}$ (σ)	SEMCD (T1) $\bar{x}$ (σ)	MD	SE	*p*	95% Confidence Interval for Difference	
						Lower Bound	Upper Bound
Intervention group (*n* = 130)	7.1 (1.6)	7.8 (1.7)	−0.742	0.144	**<0.001**	−1.026	−0.458
Comparison group (*n* = 127)	6.9 (1.8)	6.8 (1.7)	0.106	0.146	0.470	−0.182	0.394

SEMCD = Self-Efficacy for Managing Chronic Diseases 6-item Scale; T0 = baseline measurement; T1 = follow-up measurement; $\bar{x}$ = mean; σ = standard deviation; *n* = number of participants; MD = mean difference between the observed groups; SE = standard error of the mean difference between the observed groups; *p* = level of marginal significance within a statistical hypothesis test, representing the probability of the occurrence of a given event.

**Table 9:** Analysis of variance (ANOVA) with repeated measures related to physical activity self-efficacy.

	Sum of Squares	df	Mean Square	F	*p*	η^2^
PESES	2.082	1	2.082	0.222	0.638	0.001
Group: intervention, comparison	459.116	1	459.116	15.744	**<0.001**	0.059
PESES × Sex	2.692	1	2.692	0.288	0.592	0.001
PESES × Marital status	16.977	1	16.977	1.814	0.179	0.007
PESES × Household composition	3.377	1	3.377	0.361	0.549	0.001
PESES × Group: intervention, comparison	34.919	1	34.919	3.731	**0.055**	0.015
Error (PESES)	2358.327	252	9.358			
Error (Group: intervention, comparison)	7348.678	252	29.161			

PESES = Physical Exercise Self-Efficacy Scale; df = degrees of freedom; F = the ratio of the mean square for the between groups divided by the mean square within groups; *p* = level of marginal significance within a statistical hypothesis test, representing the probability of the occurrence of a given event; η^2^ = partial eta squared.

**Table 10:** Differences in expressed levels of physical activity self-efficacy between the first and second measurement time points depending on group affiliation.

Participants per Intervention	PESES (T0) $\bar{x}$ (σ)	PESES (T1) $\bar{x}$ (σ)	MD	SE	*p*	95% Confidence Interval for Difference	
						Lower Bound	Upper Bound
Intervention group (*n* = 130)	13.8 (4.5)	14.7 (4.6)	−0.886	0.391	**0.024**	−1.657	−0.115
Comparison group (*n* = 127)	12.2 (4.3)	12.0 (4.2)	0.222	0.396	0.576	−0.558	1.002

PESES = Physical Exercise Self-Efficacy Scale; T0 = baseline measurement; T1 = follow-up measurement; $\bar{x}$ = mean; σ = standard deviation; *n* = number of participants; MD = mean difference between the observed groups; SE = standard error of the mean difference between the observed groups; *p* = level of marginal significance within a statistical hypothesis test, representing the probability of the occurrence of a given event.

**Table 11:** Analysis of variance (ANOVA) with repeated measures related to nutritional self-efficacy.

	Sum of Squares	df	Mean Square	F	*p*	η^2^
NSES	71.237	1	71.237	8.617	**0.004**	0.033
Group: intervention, comparison	87.089	1	87.089	4.325	**0.039**	0.017
NSES × Sex	46.397	1	46.397	5.612	**0.019**	0.022
NSES × Marital status	2.629	1	2.629	0.318	0.573	0.001
NSES × Household composition	15.098	1	15.098	1.826	0.178	0.007
NSES × PESES_T0	38.978	1	38.978	4.715	**0.031**	0.018
NSES × Group: intervention, comparison	83.849	1	83.849	10.142	**0.002**	0.039
Error (NSES)	2075.108	251	8.267			
Error (Group: intervention, comparison)	5053.644	251	20.134			

NSES = Nutrition Self-Efficacy Scale; PESES_T0 = Physical Exercise Self-Efficacy Scale score at baseline; df = degrees of freedom; F = the ratio of the mean square for the between groups divided by the mean square within groups; *p* = level of marginal significance within a statistical hypothesis test, representing the probability of the occurrence of a given event; η^2^ = partial eta squared.

**Table 12:** Differences in expressed levels of nutritional self-efficacy between the first and second measurement time points depending on group affiliation.

Participants per Intervention	NSES (T0) $\bar{x}$ (σ)	NSES (T1) $\bar{x}$ (σ)	MD	SE	*p*	95% Confidence Interval for Difference	
						Lower Bound	Upper Bound
Intervention group (*n* = 130)	13.9 (4.2)	14.7 (4.0)	−0.984	0.370	**0.008**	−1.713	−0.254
Comparison group (*n* = 127)	12.9 (4.3)	12.4 (3.8)	0.755	0.375	**0.045**	0.016	1.493

NSES = Nutrition Self-Efficacy Scale; T0 = baseline measurement; T1 = follow-up measurement; $\bar{x}$ = mean; σ = standard deviation; *n* = number of participants; MD = mean difference between the observed groups; SE = standard error of the mean difference between the observed groups; *p* = level of marginal significance within a statistical hypothesis test, representing the probability of the occurrence of a given event.

**Table 13:** Analysis of variance (ANOVA) with repeated measures related to social support.

	Sum of Squares	df	Mean Square	F	*p*	η^2^
OSSS-3	3.288	1	3.288	1.660	0.199	0.007
Group: intervention, comparison	7.941	1	7.941	1.125	0.290	0.004
OSSS-3 × Sex	0.587	1	0.587	0.297	0.587	0.001
OSSS-3 × Marital status	1.845	1	1.845	0.931	0.335	0.004
OSSS-3 × Household composition	1.627	1	1.627	0.822	0.366	0.003
OSSS-3 × PESES_T0	2.808	1	2.808	1.418	0.235	0.006
OSSS-3 × Group: intervention, comparison	5.379	1	5.379	2.716	0.101	0.011
Error (OSSS-3)	497.108	251	1.981			
Error (Group: intervention, comparison)	1772.035	251	7.060			

OSSS-3 = Oslo Social Support Scale; PESES_T0 = Physical Exercise Self-Efficacy Scale score at baseline; df = degrees of freedom; F = the ratio of the mean square for the between groups divided by the mean square within groups; *p* = level of marginal significance within a statistical hypothesis test, representing the probability of the occurrence of a given event; η^2^ = partial eta squared.

**Table 14:** Differences in expressed levels of social support between the first and second measurement time points depending on group affiliation.

Participants per Intervention	OSSS-3 (T0) $\bar{x}$ (σ)	OSSS-3 (T1) $\bar{x}$ (σ)	MD	SE	*p*	95% Confidence Interval for Difference	
						Lower Bound	Upper Bound
Intervention group (*n* = 130)	9.5 (2.2)	10.1 (2.3)	−0.552	0.181	**0.003**	−0.909	−0.195
Comparison group (*n* = 127)	9.9 (2.1)	10.0 (2.1)	−0.112	0.184	0.543	−0.473	0.249

OSSS-3 = Oslo Social Support Scale; T0 = baseline measurement; T1 = follow-up measurement; $\bar{x}$ = mean; σ = standard deviation; *n* = number of participants; MD = mean difference between the observed groups; SE = standard error of the mean difference between the observed groups; *p* = level of marginal significance within a statistical hypothesis test, representing the probability of the occurrence of a given event.

## Data Availability

The data presented in this study are available upon request from the corresponding author. The data are not publicly available due to privacy and personal data related to Regulation (EU) 2016/679 of the European Parliament and of the Council of 27 April 2016, on the protection of individuals regarding the processing of personal data and the free movement of such data.

## Supplementary Materials

The following supporting information can be downloaded at: https://www.mdpi.com/article/10.3390/healthcare14020229/s1.

## Abbreviations

The following abbreviations are used in this manuscript:

PERMA	Positive Emotion, Engagement, Relationships, Meaning, and Accomplishment
TTM	Transtheoretical model
PHQ-8	8-item Patient Health Questionnaire Depression Scale
GSES	General Self-Efficacy Scale
SEMCD	Self-Efficacy for Managing Chronic Diseases 6-item Scale
PESES	The Physical Exercise Self-Efficacy Scale
NSES	The Nutrition Self-Efficacy Scale
OSSS-3	Oslo Social Support Scale
SPSS	Statistical Package for the Social Sciences
ISRCTN	International Standard Randomized Controlled Trial Number
GROW	Goal, Reality, Options, and Will
CDSMP	Chronic Disease Self-Management Program
GPs	General practitioners
DSM-IV	Diagnostic and Statistical Manual of Mental Disorders, Fourth Edition
SMART	Specific, Measurable, Achievable, Relevant and Time-bound
COVID-19	Coronavirus disease 2019
SEFAC	Social Engagement Framework for Addressing the Chronic Disease Challenge
T0	Baseline measurement
T1	Six months post-intervention
IBM	International Business Machines Corporation
NY	New York
USA	United States of America
ANOVA	Analysis of variance
EU	European Union

## References

[1] World Health Organization World Report on Ageing and Health. https://apps.who.int/iris/bitstream/handle/10665/186463/9789240694811_eng.pdf?sequence=1&isAllowed=y.

[2] Cojocaru L., Soponaru C., Muntele-Hendreș D., Ceobanu C. (2025). Meaning in life among aged people: A qualitative study of an institutionalized elderly sample. Eur. J. Investig. Health Psychol. Educ..

[3] Singh S., Bajorek B. (2014). Defining ‘elderly’ in clinical practice guidelines for pharmacotherapy. Pharm. Pract..

[4] World Health Organization Mental Health. https://www.who.int/news-room/fact-sheets/detail/mental-health-strengthening-our-response.

[5] Keyes C.L. (2005). Mental illness and/or mental health? Investigating axioms of the complete state model of health. J. Consult. Clin. Psychol..

[6] Fiske A., Wetherell J.L., Gatz M. (2009). Depression in older adults. Annu. Rev. Clin. Psychol..

[7] Wray N.R., Ripke S., Mattheisen M., Trzaskowski M., Byrne E.M., Abdellaoui A., Adams M.J., Agerbo E., Air T.M., Andlauer T.M.F. (2018). Genome-wide association analyses identify 44 risk variants and refine the genetic architecture of major depression. Nat. Genet..

[8] Connor-Smith J.K., Flachsbart C. (2007). Relations between personality and coping: A meta-analysis. J. Personal. Soc. Psychol..

[9] World Health Organization Mental Health of Older Adults. https://www.who.int/news-room/fact-sheets/detail/mental-health-of-older-adults.

[10] Kales H.C., Gitlin L.N., Lyketsos C.G. (2015). Assessment and management of behavioral and psychological symptoms of dementia. BMJ.

[11] World Health Organization Noncommunicable Diseases. https://www.who.int/news-room/fact-sheets/detail/noncommunicable-diseases.

[12] World Health Organization Cardiovascular Diseases (CVDs). https://www.who.int/news-room/fact-sheets/detail/cardiovascular-diseases-(cvds).

[13] International Diabetes Federation (2019). IDF Diabetes Atlas, Ninth Edition. https://diabetesatlas.org/media/uploads/sites/3/2025/02/IDF-Atlas-9th-Edition-EN.pdf.

[14] Maresova P., Javanmardi E., Barakovic S., Barakovic Husic J., Tomsone S., Krejcar O., Kuca K. (2019). Consequences of chronic diseases and other limitations associated with old age—A scoping review. BMC Public Health.

[15] Somrongthong R., Hongthong D., Wongchalee S., Wongtongkam N. (2016). The influence of chronic illness and lifestyle behaviors on quality of life among older thais. Biomed. Res. Int..

[16] Ni Z., Zhu X., Tian K., Chen Q., Yang Y., Xie S. (2024). Depressive symptoms of older adults with chronic diseases: The mediating roles of activities of daily living and economic burden of diseases. Front. Psychol..

[17] Chan S.W. (2021). Chronic disease management, self-efficacy and quality of life. J. Nurs. Res..

[18] Cheng C., Inder K., Chan S.W. (2019). Patients’ experiences of coping with multiple chronic conditions: A meta-ethnography of qualitative work. Int. J. Ment. Health Nurs..

[19] Bandura A. (1977). Self-efficacy: Toward a unifying theory of behavioral change. Psychol. Rev..

[20] Luszczynska A., Scholz U., Schwarzer R. (2005). The general self-efficacy scale: Multicultural validation studies. J. Psychol..

[21] Ong A.D., Bergeman C.S., Bisconti T.L., Wallace K.A. (2006). Psychological resilience, positive emotions, and successful adaptation to stress in later life. J. Personal. Soc. Psychol..

[22] Drewelies J., Wagner J., Tesch-Römer C., Heckhausen J., Gerstorf D. (2017). Perceived control across the second half of life: The role of physical health and social integration. Psychol. Aging.

[23] Rowe J.W., Kahn R.L. (1997). Successful aging. Gerontologist.

[24] Estebsari F., Dastoorpoor M., Khalifehkandi Z.R., Nouri A., Mostafaei D., Hosseini M., Esmaeili R., Aghababaeian H. (2020). The concept of successful aging: A review article. Curr. Aging Sci..

[25] Fountain-Zaragoza S., Prakash R.S. (2017). Mindfulness training for healthy aging: Impact on attention, well-being, and inflammation. Front. Aging Neurosci..

[26] Kabat-Zinn J. (2003). Mindfulness-Based Interventions in Context: Past, Present, and Future. Clin. Psychol..

[27] Shonin E., Van Gordon W., Griffiths M.D. (2014). Are there risks associated with using mindfulness in the treatment of psychopathology?. Clin. Pract..

[28] Goyal M., Singh S., Sibinga E.M., Gould N.F., Rowland-Seymour A., Sharma R., Berger Z., Sleicher D., Maron D.D., Shihab H.M. (2014). Meditation programs for psychological stress and well-being: A systematic review and meta-analysis. JAMA Intern. Med..

[29] Hofmann S.G., Sawyer A.T., Witt A.A., Oh D. (2010). The effect of mindfulness-based therapy on anxiety and depression: A meta-analytic review. J. Consult. Clin. Psychol..

[30] Khoury B., Sharma M., Rush S.E., Fournier C. (2015). Mindfulness-based stress reduction for healthy individuals: A meta-analysis. J. Psychosom. Res..

[31] Juraga D., Rukavina T., Marinović Glavić M., Roviš D., Racz A., Bilajac L., Antonić M., Raat H., Vasiljev V. (2025). Impact of face-to-face and online mindfulness-based public health interventions on health-related quality of life in older people: A comparative study. Int. J. Environ. Res. Public Health.

[32] Garland E.L., Geschwind N., Peeters F., Wichers M. (2015). Mindfulness training promotes upward spirals of positive affect and cognition: Multilevel and autoregressive latent trajectory modeling analyses. Front. Psychol..

[33] Shonin E., Van Gordon W., Griffiths M.D. (2015). Does mindfulness work?. BMJ.

[34] Li S.Y.H., Bressington D. (2019). The effects of mindfulness-based stress reduction on depression, anxiety, and stress in older adults: A systematic review and meta-analysis. Int. J. Ment. Health Nurs..

[35] Bein M., Lesage M., Dikaios E., Chakravarty M., Segal Z., Royal I., Speechley M., Schiavetto A., Blumberger D., Sacchet M.D. (2022). Mindfulness-based cognitive therapy vs. a health enhancement program for the treatment of late-life depression: Study protocol for a multi-site randomized controlled trial. Front. Aging Neurosci..

[36] Scott-Sheldon L.A.J., Gathright E.C., Donahue M.L., Balletto B., Feulner M.M., DeCosta J., Cruess D.G., Wing R.R., Carey M.P., Salmoirago-Blotcher E. (2020). Mindfulness-Based Interventions for Adults with Cardiovascular Disease: A Systematic Review and Meta-Analysis. Ann. Behav. Med..

[37] Ee C.C., Al-Kanini I., Armour M., Piya M.K., McMorrow R., Rao V.S., Naidoo D., Metzendorf M.I., Kroeger C.M., Sabag A. (2025). Mindfulness-based interventions for adults with type 2 diabetes mellitus: A systematic review and meta-analysis. Integr. Med. Res..

[38] Whitmore J. (2009). Coaching for Performance: GROWing Human Potential and Purpose—The Principles and Practice of Coaching and Leadership.

[39] Tan S.S., Pisano M.M., Boone A.L., Baker G., Pers Y.M., Pilotto A., Valsecchi V., Zora S., Zhang X., Fierloos I. (2019). Evaluation Design of EFFICHRONIC: The Chronic Disease Self-Management Programme (CDSMP) Intervention for Citizens with a Low Socioeconomic Position. Int. J. Environ. Res. Public Health.

[40] Mindfulness Association Mindfulness Based Living Course (MBLC). https://www.mindfulnessassociation.net/mindfulness-courses/8-week-mblc/.

[41] Handley M.A., Lyles C.R., McCulloch C., Cattamanchi A. (2018). Selecting and improving quasi-experimental designs in effectiveness and implementation research. Annu. Rev. Public Health.

[42] Dweck C.S., Yeager D.S. (2019). Mindsets: A view from two eras. Perspect. Psychol. Sci..

[43] Bailey R.R. (2017). Goal setting and action planning for health behavior change. Am. J. Lifestyle Med..

[44] Mittelmark M.B., Bauer G.F., Mittelmark M.B., Bauer G.F., Vaandrager L., Pelikan J.M., Sagy S., Eriksson M., Lindström B., Meier Magistretti C. (2022). Salutogenesis as a Theory, as an Orientation and as the Sense of Coherence. The Handbook of Salutogenesis.

[45] Coulter A., Oldham J. (2016). Person-centred care: What is it and how do we get there?. Future Hosp. J..

[46] Kovich M.K., Simpson V.L., Foli K.J., Hass Z., Phillips R.G. (2023). Application of the PERMA model of well-being in undergraduate students. Int. J. Community Wellbeing.

[47] Abrash Walton A., Nageotte N.L., Heimlich J.E., Threadgill A.V. (2022). Facilitating behavior change: Introducing the Transtheoretical Model of Behavior Change as a conservation psychology framework and tool for practitioners. Zoo Biol..

[48] Brooks P.J., Ripoll P., Sánchez C., Torres M. (2023). Coaching leaders toward favorable trajectories of burnout and engagement. Front. Psychol..

[49] Juraga D., Rukavina T., Bilajac L., Marinović Glavić M., Roviš D., Raat H., Vasiljev V. (2022). Comparison of Conventional (Face-To-Face) and Online Approach in Mindfulness-Based Chronic Disease Self-Management Interventions for Older Adults. J. Public Health Res..

[50] Leong S.M., Lei W.I., Chan U.W. (2020). The six-month and one-year outcome of a chronic disease self-management program among older adults in Macao: A quasi-experimental study. SAGE Open Nurs..

[51] Gallegos A.M., Moynihan J., Pigeon W.R. (2018). A secondary analysis of sleep quality changes in older adults from a randomized trial of an MBSR program. J. Appl. Gerontol..

[52] Zhang X., Tan S.S., Fierloos I., Zanutto O., Alhambra-Borrás T., Vasiljev V., Bennett S., Rentoumis T., Buranello A., Macchione S. (2019). Evaluation Design of the Social Engagement Framework for Addressing the Chronic-Disease-Challenge (SEFAC): A Mindfulness-Based Intervention to Promote the Self-Management of Chronic Conditions and a Healthy Lifestyle. BMC Public Health.

[53] Sigudla J., Maritz J.E. (2023). Exploratory factor analysis of constructs used for investigating research uptake for public healthcare practice and policy in a resource-limited setting, South Africa. BMC Health Serv. Res..

[54] Kroenke K., Strine T.W., Spitzer R.L., Williams J.B., Berry J.T., Mokdad A.H. (2009). The PHQ-8 as a measure of current depression in the general population. J. Affect. Disord..

[55] Hu H., Li G., Arao T. (2013). Validation of a Chinese version of the self-efficacy for managing chronic disease 6-item scale in patients with hypertension in primary care. Int. Sch. Res. Not..

[56] Schwarzer R., Renner B. Health-Specific Self-Efficacy Scales. https://userpage.fu-berlin.de/health/healself.pdf.

[57] Dimitrov N., Brähler E., Hering T., Glaesmer H., Zenger M. (2025). Normative values and psychometric properties of the Oslo Social Support Scale-3 (OSSS-3) for adults aged 60 to 85 years. Eur. J. Ageing.

[58] Zhang D., Yuan T., Huang A., Li X., Yang L., Wang C., Liu M., Lei Y., Sun L., Li J. (2024). Validation of the Chinese version of the Oslo-3 Social Support Scale among nursing students: A study based on Classical Test Theory and Item Response Theory models. BMC Nurs..

[59] Remm S.E., Halcomb E., Peters K., Hatcher D., Frost S.A. (2023). Self-efficacy, resilience and healthy ageing among older people who have an acute hospital admission: A cross-sectional study. Nurs. Open.

[60] Hladek M.D., Reimer T., Casey J., Nkimbeng M., Peeler A., Nelson K.E., Taylor J., Han H.R., Szanton S.L. (2025). Measuring self-efficacy in U.S. older adults with chronic disease: A systematic review. Aging Ment. Health.

[61] Li X., Yang K., An Y., Liu M., Yan C., Huang R. (2022). General self-efficacy and frailty in hospitalized older patients: The mediating effect of loneliness. Geriatr. Nurs..

[62] Kerari A., Bahari G., Alharbi K., Alenazi L. (2024). The effectiveness of the chronic disease self-management program in improving patients’ self-efficacy and health-related behaviors: A quasi-experimental study. Healthcare.

[63] Woo S.H., Seo J.P., Kim H.R., So W.Y., Sim Y.K. (2024). Health-promoting behaviors, physical self-efficacy, exercise adherence, and sports commitment among older adults who participate in sports activities. Healthcare.

[64] Gilcharan Singh H.K., Chee W.S.S., Hamdy O., Mechanick J.I., Lee V.K.M., Barua A., Mohd Ali S.Z., Hussein Z. (2020). Eating self-efficacy changes in individuals with type 2 diabetes following a structured lifestyle intervention based on the transcultural Diabetes Nutrition Algorithm (tDNA): A secondary analysis of a randomized controlled trial. PLoS ONE.

[65] Guicciardi M., Lecis R., Anziani C., Corgiolu L., Porru A., Pusceddu M., Spanu F. (2014). Type 2 diabetes mellitus, physical activity, exercise self-efficacy, and body satisfaction. An application of the transtheoretical model in older adults. Health Psychol. Behav. Med..

[66] Maher J.M., Markey J.C., Ebert-May D. (2013). The other half of the story: Effect size analysis in quantitative research. CBE Life Sci. Educ..

[67] Bakeman R. (2005). Recommended effect size statistics for repeated measures designs. Behav. Res. Methods.

[68] Des Jarlais D.C., Lyles C., Crepaz N., TREND Group (2004). Improving the Reporting Quality of Nonrandomized Evaluations of Behavioral and Public Health Interventions: The TREND Statement. Am. J. Public Health.

[69] Hunter J.B., Fernandez M.L., Lacy-Martinez C.R., Dunne-Sosa A.M., Coe M.K. (2007). Male preventive health behaviors: Perceptions from men, women, and clinical staff along the U.S. Mexico border. Am. J. Men’s Health.

[70] Sanchez D. Men are More Reluctant to Go to the Doctor—And it’s Putting Them at Risk. https://www.weforum.org/agenda/2020/06/men-healthcare-cancer-heart-disease/#:~:text=Jun%203%2C%202020%20This%20article,Psychology%2C%20Rutgers%20University%20Our%20Impact.

[71] Heidelbaugh J.J. (2018). The adult well-male examination. Am. Fam. Physician.

[72] Novak J.R., Peak T., Gast J., Arnell M. (2019). Associations between masculine norms and health-care utilization in highly religious, heterosexual men. Am. J. Men’s Health.

[73] Wang Y., Hunt K., Nazareth I., Freemantle N., Petersen I. (2013). Do men consult less than women? An analysis of routinely collected UK general practice data. BMJ Open.

[74] Courtenay W.H. (2000). Constructions of masculinity and their influence on men’s well-being: A theory of gender and health. Soc. Sci. Med..

[75] Lowe H., Cheung G. (2023). Old age psychiatry. Australas. Psychiatry.

[76] Doménech S., Blancafort-Alias S., Rojano X., Salvà A., Roqué M., Coll-Planas L. (2023). Subjective psychological impacts during COVID-19 lockdown on older people, risk profiles and coping strategies: Results of an online survey in Spain. J. Community Psychol..

[77] Long L., Ji D., Hu C., Yang L., Tang S., Wang Y. (2023). Microneedles for in situ tissue regeneration. Mater. Today Bio.

[78] Figueira H.A., Figueira O.A., Figueira A.A., Figueira J.A., Polo-Ledesma R.E., Lyra da Silva C.R., Dantas E.H.M. (2023). Impact of physical activity on anxiety, depression, stress and quality of life of the older people in Brazil. Int. J. Environ. Res. Public Health.

[79] Earl E.J., Marais D. (2023). The experience of intergenerational interactions and their influence on the mental health of older people living in residential care. PLoS ONE.

[80] Nielsen M.W., Stefanick M.L., Peragine D., Neilands T.B., Ioannidis J.P.A., Pilote L., Prochaska J.J., Cullen M.R., Einstein G., Klinge I. (2021). Gender-related variables for health research. Biol. Sex Differ..

[81] Vuletić G., Stapić M. (2013). Kvaliteta života i doživljaj usamljenosti kod osoba starije životne dobi. Klin. Psihol..

[82] Hudgens S., Floden L., Blackowicz M., Jamieson C., Popova V., Fedgchin M., Drevets W.C., Cooper K., Lane R., Singh J. (2021). Meaningful change in depression symptoms assessed with the Patient Health Questionnaire (PHQ-9) and Montgomery-Åsberg Depression Rating Scale (MADRS) among patients with treatment resistant depression in two, randomized, double-blind, active-controlled trials of Esketamine nasal spray combined with a new oral antidepressant. J. Affect. Disord..

[83] Creswell J.D. (2017). Mindfulness interventions. Annu. Rev. Psychol..

[84] Young L.A., Cappola A.R., Baime M.J. (2009). Mindfulness based stress reduction: Effect on emotional distress in diabetes. Pract. Diabetes Int..

[85] Guendelman S., Medeiros S., Rampes H. (2017). Mindfulness and emotion regulation: Insights from neurobiological, psychological, and clinical studies. Front. Psychol..

[86] Phillips M.L., Drevets W.C., Rauch S.L., Lane R. (2003). Neurobiology of emotion perception II: Implications for major psychiatric disorders. Biol. Psychiatry.

[87] Kangas J.L., Baldwin A.S., Rosenfield D., Smits J.A., Rethorst C.D. (2015). Examining the moderating effect of depressive symptoms on the relation between exercise and self-efficacy during the initiation of regular exercise. Health Psychol..

[88] Gold A.K., Rabideau D.J., Katz D., Peters A.T., Bist J., Albury E.A., George N., Hsu I.R., Faulkner M., Pletcher M.J. (2024). Self-efficacy for exercise in adults with lifetime depression and low physical activity. Psychiatry Res. Commun..

[89] Chair S.Y., Cheng H.Y., Chew H.S.J., Zang Y.L., Siow E.K.C., Cao X. (2020). Leisure-time physical activity and depressive symptoms among patients with coronary heart disease: The mediating role of physical activity self-efficacy. Worldviews Evid. Based Nurs..

[90] Chandna S., Sharma P., Moosath H. (2022). The mindful self: Exploring mindfulness in relation with self-esteem and self-efficacy in Indian population. Psychol. Stud..

[91] Bayır B., Aylaz R. (2021). The effect of mindfulness-based education given to individuals with substance-use disorder according to self-efficacy theory on self-efficacy perception. Appl. Nurs. Res..

[92] Tan J., Yang W., Ma H., Yu Y. (2016). Adolescents’ core self-evaluations as mediators of the effect of mindfulness on life satisfaction. Soc. Behav. Pers..

[93] Khodarahimi S. (2010). General self-efficacy and worry in an Iranian adolescents and youths samples. Educ. Res..

[94] Ross-Stewart L., Short S.E. (2009). The frequency and perceived effectiveness of images used to build, maintain, and regain confidence. J. Appl. Sport Psychol..

[95] de Jong A., Hommes M., Brouwers A., Tomic W. (2013). Effects of mindfulness-based stress reduction course on stress, mindfulness, job self-efficacy and motivation among unemployed people. Aust. J. Career Dev..

[96] Lorig K.R., Sobel D.S., Ritter P.L., Laurent D., Hobbs M. (2001). Effect of a self-management program on patients with chronic disease. Eff. Clin. Pract..

[97] Tang Y.Y., Hölzel B.K., Posner M.I. (2015). The neuroscience of mindfulness meditation. Nat. Rev. Neurosci..

[98] Korenhof S.A., Rouwet E.V., Elstgeest L.E.M., Tan S.S., Macchione S., Vasiljev V., Rukavina T., Alhambra-Borrás T., Fierloos I.N., Raat H. (2022). Evaluation of an intervention to promote self-management regarding cardiovascular disease: The Social Engagement Framework for Addressing the Chronic-Disease-Challenge (SEFAC). Int. J. Environ. Res. Public Health.

[99] Sagui-Henson S.J., Levens S.M., Blevins C.L. (2018). Examining the psychological and emotional mechanisms of mindfulness that reduce stress to enhance healthy behaviours. Stress Health.

[100] Vago D.R., Silbersweig D.A. (2012). Self-awareness, self-regulation, and self-transcendence (S-ART): A framework for understanding the neurobiological mechanisms of mindfulness. Front. Hum. Neurosci..

[101] Hölzel B.K., Lazar S.W., Gard T., Schuman-Olivier Z., Vago D.R., Ott U. (2011). How does mindfulness meditation work? Proposing mechanisms of action from a conceptual and neural perspective. Perspect. Psychol. Sci..

[102] Baer R.A. (2003). Mindfulness training as a clinical intervention: A conceptual and empirical review. Clin. Psychol. Sci. Pract..

[103] Arch J.J., Craske M.G. (2006). Mechanisms of mindfulness: Emotion regulation following a focused breathing induction. Behav. Res. Ther..

[104] Gidugu V., Jacobs M.L. (2019). Empowering individuals with mental illness to develop healthy eating habits through mindful eating: Results of a program evaluation. Psychol. Health Med..

[105] Kennedy L.E., Misyak S., Hosig K., Duffey K.J., Ju Y., Serrano E. (2018). The slow down program: A mixed methods pilot study of a mindfulness-based stress management and nutrition education program for mothers. Complement. Ther. Med..

[106] Stephens J.D., Althouse A., Tan A., Melnyk B.M. (2017). The role of race and gender in nutrition habits and self-efficacy: Results from the young adult weight loss study. J. Obes..

[107] Schwarzer R., Warner L.M., Fleig L., Gholami M., Serra-Majem L., Ngo J., Cianferotti L., Kritikou M., Mossi P., Ntzani E. (2018). Dietary planning, self-efficacy, and outcome expectancies play a role in an online intervention on fruit and vegetable consumption. Psychol. Health.

[108] Kocalevent R.D., Berg L., Beutel M.E., Hinz A., Zenger M., Härter M., Nater U., Brähler E. (2018). Social support in the general population: Standardization of the Oslo social support scale (OSSS-3). BMC Psychol..

[109] Uchino B.N. (2009). Understanding the links between social support and physical health: A life-span perspective with emphasis on the separability of perceived and received support. Perspect. Psychol. Sci..

[110] Wrzus C., Hänel M., Wagner J., Neyer F.J. (2013). Social network changes and life events across the life span: A meta-analysis. Psychol. Bull..

[111] Freedman V.A., Hu M., Kasper J.D. (2022). Changes in older adults’ social contact during the COVID-19 pandemic. J. Gerontol. B. Psychol. Sci. Soc. Sci..

[112] Verhaeghen P., Aikman S.N., Mirabito G. (2025). Mindfulness interventions in older adults for mental health and well-being: A meta-analysis. J. Gerontol. B. Psychol. Sci. Soc. Sci..

[113] Lindsay E.K., Young S., Brown K.W., Smyth J.M., Creswell J.D. (2019). Mindfulness training reduces loneliness and increases social contact in a randomized controlled trial. Proc. Natl. Acad. Sci. USA.

[114] Kirkland S.A., Griffith L.E., Oz U.E., Thompson M., Wister A., Kadowaki L., Basta N.E., McMillan J., Wolfson C., Raina P. (2023). Increased prevalence of loneliness and associated risk factors during the COVID-19 pandemic: Findings from the Canadian Longitudinal Study on Aging (CLSA). BMC Public Health.

[115] Kung C.S.J., Steptoe A. (2024). Changes in well-being among socially isolated older people during the COVID-19 pandemic: An outcome-wide analysis. Proc. Natl. Acad. Sci. USA.

[116] Schuman-Olivier Z., Trombka M., Lovas D.A., Brewer J.A., Vago D.R., Gawande R., Dunne J.P., Lazar S.W., Loucks E.B., Fulwiler C. (2020). Mindfulness and behavior change. Harv. Rev. Psychiatry.

[117] Alvarado-García P.A.A., Soto-Vásquez M.R., Infantes Gomez F.M., Guzman Rodriguez N.M., Castro-Paniagua W.G. (2025). Effect of a mindfulness program on stress, anxiety, depression, sleep quality, social support, and life satisfaction: A quasi-experimental study in college students. Front. Psychol..

[118] Keng S.L., Smoski M.J., Robins C.J. (2011). Effects of mindfulness on psychological health: A review of empirical studies. Clin. Psychol. Rev..

[119] Mollborn S., Lawrence E.M., Hummer R.A. (2020). A gender framework for understanding health lifestyles. Soc. Sci. Med..

[120] Nowak P.F., Rogowska A.M., Kwaśnicka A. (2024). The mediating role of health behaviors in the relationship between internal locus of control and life satisfaction in public health students. Sci. Rep..

[121] Patrão A.L., Almeida M.D.C., Matos S.M.A., Chor D., Aquino E.M.L. (2017). Gender and psychosocial factors associated with healthy lifestyle in the Brazilian Longitudinal Study of Adult Health (ELSA-Brasil) cohort: A cross-sectional study. BMJ Open.

