# Supplementary material for: Improving Mental Health, Self-Efficacy and Social Support in Older People Through Community Intervention Based on Mindfulness: A Quasi-Experimental Study
Source: Healthcare (Basel). 2026 Jan 16;14(2):229. doi: 10.3390/healthcare14020229 (PMC12840687; doi:10.3390/healthcare14020229)
Supplement: Supplementary file 1 [file healthcare-14-00229-s001.zip › healthcare-4003789-supplementary.pdf]

Denis Juraga, PhD
Department of Social Medicine and Epidemiology
Faculty of Medicine, University of Rijeka
Braće Branchetta 20
51000, Rijeka
denis.juraga@uniri.hr

Special Issue Editors
Healthcare
“Public Health Prevention Through Integrative Medicine: Community-Based and Society-Level Interventions" Special Issue

SUPPLEMENTARY MATERIAL

Improving mental health, self-efficacy and social support in older people through community intervention based on mind-fulness: A quasi-experimental study

Corresponding author: Denis Juraga, Department of Social Medicine and Epidemiology, Faculty of Medicine, University of Rijeka, Braće Branchetta 20/1, 51000, Rijeka, Primorje-Gorski Kotar County, Croatia. Tel. +385-51-651-220 / +385-91-581-1632. Email: denis.juraga@medri.uniri.hr

1. Detailed description of the theoretical framework of the seven-week mindfulness-based community program

The theoretical framework of the seven-week mindfulness-based community program consisted of the following segments: the integrated theoretical framework of salutogenesis (Antonovsky’s theory) guided the participants to focus on enhancing their “sense of coherence” and internal resources for well-being, rather than merely addressing disease or deficits. This meant that the mindfulness-based workshops were framed positively, helping older people identify meaningful aspects of life, build coping resources, and strengthen overall well-being [44]. The workshops were also framed within the person-cantered approach as the program was delivered in an encouraging, non-judgmental manner and tailored to respect each participant’s individual needs [45]. The positive psychology concept, based on the Seligman’s PERMA model (Positive Emotion, Engagement, Relationships, Meaning, and Accomplishment) was also integrated within the workshops as mindfulness practices cultivated positive emotions, reflective activities promoted deep engagement in the present moment, group-based sessions encouraged supportive relationships among participants, discussions on values and life purpose helped participants find meaning in their experience and built in goal-setting and feedback loops helped the participants to have a sense of achievement [46]. The Transtheoretical Model (TTM) shaped the intervention structure and tailor strategies to participants’ readiness for behaviour change through five stages: Precontemplation, Contemplation, Preparation, Action,

and Maintenance [47]. The design of the program incorporated content to meet older people at their respective stages and to facilitate progression through these stages. The GROW model (Goal, Reality, Options and Will model) from coaching psychology was used to address the facilitation style and interactive elements of the goal-oriented intervention. The GROW framework is a flexible four-stage coaching process in which the facilitator guides individuals to set a Goal, examine their current Reality, explore Options, and commit to a course of action or Will [48].
